# Phosphatidylinositol phosphates modulate interactions between the StarD4 sterol trafficking protein and lipid membranes

**DOI:** 10.1016/j.jbc.2022.102058

**Published:** 2022-05-20

**Authors:** Xiaoxue Zhang, Hengyi Xie, David Iaea, George Khelashvili, Harel Weinstein, Frederick R. Maxfield

**Affiliations:** 1 Department of Biochemistry, Weill Cornell Medical College, New York, New York, USA; 2 Department of Physiology and Biophysics, Weill Cornell Medical College, New York, New York, USA; 3 Weill Cornell Medical College, Rockefeller University, and Memorial Sloan-Kettering Cancer Center Tri-Institutional Chemical Biology Program, New York, New York, USA

**Keywords:** cholesterol-binding protein, electron microscopy, computer modeling, membrane lipid, liposome, lipid transport, ACAT, acyl-CoA:cholesterol acyl-transferase, dansyl-PE, dansyl-phosphatidylethanolamine, DHE, dehydroergosterol, DLS, dynamic light scattering, ER, endoplasmic reticulum, ERC, endocytic recycling compartment, MFM, mean-field model, NBD-DOPE, 1,2-dioleoyl-sn-glycero-3-phosphoethanolamine-N-(7-nitro-2–1,3-benzoxadiazol-4-yl), PI(3,5)P_2_, phosphatidylinositol 3,5-bisphosphate, PI(4)P, phosphatidylinositol 4-phosphate, PI(4,5)P_2_, phosphatidylinositol 4,5-bisphosphate, PI, phosphatidylinositol, PIP, phosphatidylinositol phosphate, PIP_2_, phosphatidylinositol bisphosphate, POPC, 1-palmitoyl-2-oleoyl-glycero-3-phosphocholine, POPE, 1-palmitoyl-2-oleoyl-sn-glycero-3-phosphoethanolamine, POPS, 1-palmitoyl-2-oleoyl-sn-glycero-3-phospho-L-serine, PS, phosphatidylserine, Rho-DOPE, 1,2-dioleoyl-sn-glycero-3-phosphoethanolamine-N-(lissamine rhodamine B sulfonyl), StarT, steroidogenic acute regulatory protein-related lipid transfer

## Abstract

There is substantial evidence for extensive nonvesicular sterol transport in cells. For example, lipid transfer by the steroidogenic acute regulator-related proteins (StarD) containing a StarT domain has been shown to involve several pathways of nonvesicular trafficking. Among the soluble StarT domain–containing proteins, StarD4 is expressed in most tissues and has been shown to be an effective sterol transfer protein. However, it was unclear whether the lipid composition of donor or acceptor membranes played a role in modulating StarD4-mediated transport. Here, we used fluorescence-based assays to demonstrate a phosphatidylinositol phosphate (PIP)-selective mechanism by which StarD4 can preferentially extract sterol from liposome membranes containing certain PIPs (especially, PI(4,5)P_2_ and to a lesser degree PI(3,5)P2). Monophosphorylated PIPs and other anionic lipids had a smaller effect on sterol transport. This enhancement of transport was less effective when the same PIPs were present in the acceptor membranes. Furthermore, using molecular dynamics (MD) simulations, we mapped the key interaction sites of StarD4 with PIP-containing membranes and identified residues that are important for this interaction and for accelerated sterol transport activity. We show that StarD4 recognizes membrane-specific PIPs through specific interaction with the geometry of the PIP headgroup as well as the surrounding membrane environment. Finally, we also observed that StarD4 can deform membranes upon longer incubations. Taken together, these results suggest a mechanism by which PIPs modulate cholesterol transfer activity *via* StarD4.

Sterols are critical components of eukaryotic cell membranes. Cholesterol is heterogeneously distributed among cellular organelles with ∼60% of total cellular cholesterol in the plasma membrane and relatively low amounts in the mitochondria and endoplasmic reticulum (ER), the latter being the site of cholesterol sensing, biosynthesis, and esterification (1, 2, 3, 4, 5). The endocytic recycling compartment (ERC) has been shown to be a major pool of intracellular cholesterol in several cell types (6, 7, 8).

Cholesterol can move between membranes by vesicular and nonvesicular transport mechanisms (3, 9). Like other lipids, cholesterol can be incorporated into transport vesicles that carry membrane components between organelles (3, 10, 11). Yet, only a small fraction of internalized plasma membrane lipids reach the ER, indicating that cholesterol sensing in the ER would be very slow and inefficient if cholesterol trafficking depended solely on vesicular transport. Indeed, there is substantial evidence for high rates of nonvesicular sterol transport in cells (3, 8, 10, 11). Several protein families have been characterized as sterol transfer proteins, meaning that they are capable of transferring sterols between membranes (12, 13, 14, 15, 16). One major family of sterol transfer proteins that have been implicated in such trafficking is the steroidogenic acute regulatory protein (StAR)-related lipid-transfer (StarT) domain family (7, 14).

The mammalian StarT domain protein family is composed of 15 members that group into six subfamilies based on domain architecture and ligand specificity (13, 14, 16, 17). Among the soluble StarT domain proteins, StarD4 has been shown to facilitate cholesteryl ester accumulation in lipid droplets in an acyl-CoA:cholesterol acyl-transferase (ACAT)-dependent manner (6, 7, 18), and it is transcriptionally regulated by SREBP-2 (7, 19).

Human StarD4 consists of 205 amino acids that fold into an α/β helix-grip structure, containing a pocket for sterol binding (20, 21, 22, 23, 24). StarD4 is well adapted to transfer sterol (22, 23) and has been shown to move sterol between synthetic model membranes (7, 23). This transfer activity involves sequential transient interaction with two membrane compartments, one membrane compartment acting as the donor and the other as the acceptor (3, 7, 23). As such, StarD4 may function as either a nonselective equilibrator or a vectorial transporter that responds to differences in lipid composition of membranes (25).

As a nonselective equilibrator, StarD4 would transfer sterol between cellular organelles based on the sterol’s chemical activity in the different membrane compartments (10, 11). StarD4’s sterol transfer activity is, however, in part mediated by a basic region that interacts with anionic lipids such as phosphatidylserine (PS) (7, 22, 23). This suggests that there may be selective targeting of StarD4 to membranes mediated by anionic lipids. PS itself is unlikely to be a useful targeting lipid because it is widely distributed among cellular organelles (26, 27, 28), making it difficult to envision how StarD4 could recognize cellular organelles as either donor or acceptor based solely on PS.

By manipulating StarD4 levels in cells, we have shown that it plays an important role in transporting sterols between the plasma membrane and the ERC (7, 29). There was about a 25% reduction in this sterol transport when StarD4 was knocked down by shRNA (29). Increases in StarD4 expression increase sterol delivery to the ER (7). These previous results show that StarD4 can influence sterol transport between multiple organelles.

Recently, the activities of two sterol transport proteins were found to be modulated by phosphatidylinositol 4-phosphate (PI(4)P) in a membrane-specific manner (30, 31). As specific phosphatidylinositol phosphates (PIPs) are selectively enriched on cellular organelles (32), they can act as organelle-specific signals to target and modulate soluble sterol transporters.

Here, we utilize a combination of biochemical, biophysical, and computational techniques to explore the effects of PIPs on StarD4 activity and the effects of StarD4 on the morphology of membranes containing various PIPs. With experimental assays of sterol transfer between such vesicles and computational molecular dynamics (MD) simulations of StarD4–membrane interactions, we map the PIP-interaction sites on StarD4 and show that the PIP membrane composition affects the mode of binding of StarD4 and the sterol transfer rate. The results of these studies point toward a mechanism of rapid vectorial transport in which StarD4 extracts sterol from donor membranes mimicking plasma membranes containing phosphatidylinositol 4,5-bisphosphate (PI(4,5)P_2_) and delivers sterol to acceptor membranes mimicking ER. The results also show that StarD4 can induce deformation in donor membranes containing PIPs, but no change in morphology is observed in acceptor membranes containing only PS as an anionic lipid.

## Results

### PIPs increase the rate of sterol transfer by StarD4 between liposomes

Murine StarD4 has been shown to transfer the fluorescent sterol, dehydroergosterol (DHE), between anionic donor and acceptor membranes (7, 23). As starting compositions for testing the effects of PIPs, we used liposomes that mimic the cytoplasmic leaflet of the plasma membrane as donors and liposomes that mimic the ER membrane as acceptors (7, 23, 28). The anionic lipid components in donor and acceptor liposomes were PS (23 mol %) and phosphatidylinositol (PI/PS, 15/5 mol %), respectively. We examined the effects of PIPs by replacing all the anionic lipids from either donor or acceptor with 0.5, 1, 2, 5, 10 mol % of PI(4,5)P_2_ or phosphatidylinositol 3,5-bisphosphate (PI(3,5)P_2_). The rest of the anionic lipids were replaced by the zwitterionic lipid phosphatidylcholine so that the PIPs are the only anionic lipids in the PIP-containing liposomes. Control liposomes prepared with the same percentages of PS were also tested in comparisons. The sterol transfer rates were measured as described previously (7). Donor liposomes containing the fluorescent cholesterol analog, DHE, and acceptor liposomes containing dansyl-phosphatidylethanolamine (dansyl-PE) are mixed with purified WT StarD4. The delivery of sterol from donor to acceptor liposomes results in a sensitized FRET signal when the two fluorescent lipids are in the same liposome.

**Figure 1:**
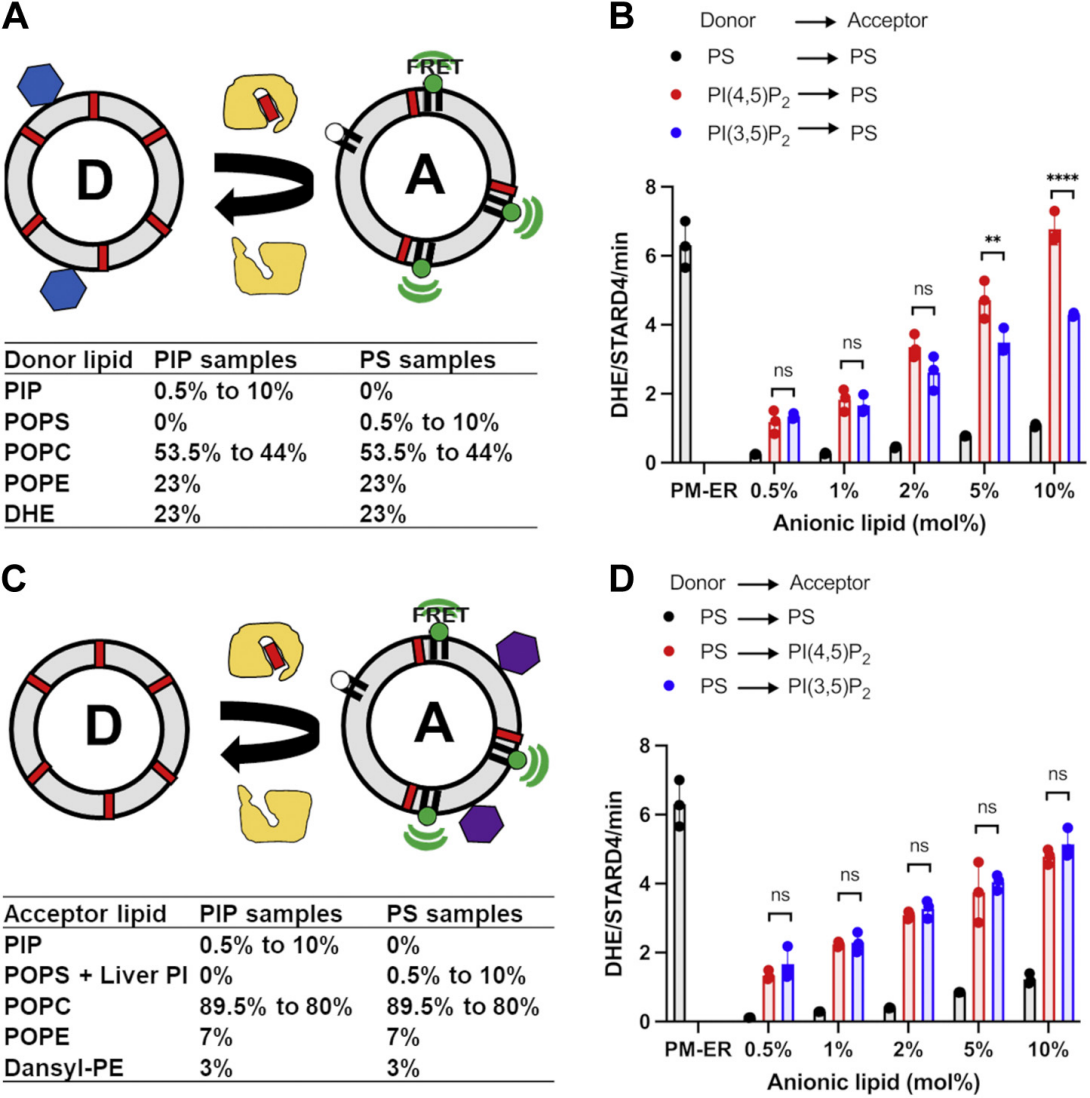
**Addition of either PI(4,5)P**_**2**_**or PI(3,5)P**_**2**_**to donor or acceptor liposomes increases the rate of StarD4-mediated sterol transfer between donor and acceptor liposomes**. *A*, illustration of model system for sterol transport with varying lipid composition in donor liposomes and a constant acceptor liposome composition. *B*, quantification of the number of DHE molecules transferred per molecule of StarD4 per minute using PI(3,5)P_2_ or PI(4,5)P_2_ in donor liposomes compared with liposomes that contain the same amount of POPS. Lipid compositions are shown in (*A*). *C*, illustration of model system for sterol transport with varying lipid composition in acceptor liposomes and a constant donor liposome composition. *D*, quantification of the number of DHE molecules transferred per molecule of StarD4 per minute using PI(3,5)P_2_ or PI(4,5)P_2_ in acceptor liposomes compared with liposomes which contain same amount of anionic lipids. Lipid compositions are shown in table. POPS or a PIP were the sole anionic lipids in donor liposomes. Control donor liposomes mimic the cytoplasmic leaflet of the plasma membrane (23 mol % POPS, 31 mol % POPC, 23 mol % POPE, 23 mol % DHE), and control acceptor liposomes mimic ER membranes (5 mol % POPS, 15 mol % liver-PI, 70 mol % POPC, 7 mol % POPE, 3 mol % dansyl-PE). For PIP-containing donor liposomes, all anionic lipids (POPS and Liver-PI) were removed and 0.5, 1, 2, 5, 10 mol % of PI(3,5)P_2_ (*red*) or PI(4,5)P_2_ (*blue*) was added to donor or acceptor, the rest of the anionic lipids were replaced by POPC. Samples containing the same fraction of POPS (*black*) were also tested. Experiments were conducted in HK buffer (50 mM Hepes, 120 mM potassium acetate, pH 7.2) at 37 °C. Hundred micromolar donor and hundred micromolar acceptor liposomes (total lipid concentration) were incubated with 1 μM WT StarD4 from time zero. Data are plotted from the average of three independent experiments ± SE. ∗∗*p* < 0.01; ∗∗∗∗*p* < 0.0001; ns, non-significant. A summary of statistical significance for this figure is shown in Table S1. ER, endoplasmic reticulum; PIP, phosphatidylinositol phosphate; PI(4,5)P2, phosphatidylinositol 4,5-bisphosphate; DHE, dehydroergosterol; PI, phosphatidylinositol; PI(3,5)P2, phosphatidylinositol 3,5-bisphosphate; dansyl-PE, dansyl-phosphatidylethanolamine; POPC, 1-palmitoyl-2-oleoyl-glycero-3-phosphocholine; POPE, 1-palmitoyl-2-oleoyl-sn-glycero-3-phosphoethanolamine; POPS, 1-palmitoyl-2-oleoyl-sn-glycero-3-phospho-L-serine.

Utilizing 23 mol % PS-containing control liposomes, StarD4 transfers seven molecules DHE/molecule StarD4/minute between donor and acceptor (7, 23). When PIPs were the only anionic lipids in either donor or acceptor liposomes, the sterol transfer activity of StarD4 was modulated, and the effect was concentration dependent (Fig. 1). The rate of StarD4-mediated sterol transfer increased ∼five-fold when 0.5 to 2 mol PI(4,5)P_2_ or PI(3,5)P_2_ was added to the donor liposomes as compared to the same amount of PS. The effects of low amounts of PI(4,5)P_2_ or PI(3,5)P_2_ on the sterol transfer rates were generally comparable, but as the percentage of phosphatidylinositol bisphosphate (PIP_2_) in donor liposomes increased to 5 mol % or more, PI(4,5)P_2_ exhibited larger effects on sterol transfer rate than PI(3,5)P_2_ (Fig. 1*B* and Table S1). The data reveals that when PIP_2_ reached 10 mol % in donor liposomes, the rate of StarD4-mediated sterol transport was increased 6.5-fold compared to PS by PI(4,5)P_2_, while the rate increased only four-fold by the same amount of PI(3,5)P_2._ The sterol transfer rate in the presence of 10 mol % PI(4,5)P_2_ was comparable to that of the donor control (Fig. 1*B*), which contains 23 mol % PS. It should be noted that the PI(4,5)P_2_ contains a much greater negative charge than PS. However, PI(3,5)P_2_ carries the same negative charge as PI(4,5)P_2_, and its effect on transport is less than PI(4,5)P_2_. Thus, more than charge is responsible for the specific effects of PI(4,5)P_2_.

We also examined the effects of varying the amount of PIP in the acceptor liposomes. The StarD4 activity increased 4.5-fold relative to PS-containing liposomes at all anionic lipid concentrations. Substitution of PI(4,5)P_2_ for PI(3,5)P_2_ in acceptor liposomes led to no significant differences in sterol transfer rates (Fig. 1*D* and Table S1).

### Key interaction sites of StarD4 with PIP-containing membranes underlie the spatial configurations of the protein–membrane interaction

In previous work, the StarD4–membrane interaction at a polybasic patch identified in the crystal structure of StarD4 was shown to be essential for the function of StarD4 (22, 23). Thus, the mutation of either K49, K52, or K219 in the basic patch to alanine strongly hampered the sterol transfer kinetics of StarD4 on PS-containing membrane (23). We reasoned, therefore, that the specificity for the kinds of PIP lipids contained in the membrane, and the PIP-dependent regulation of kinetics, are also likely to be mediated by these lipids interacting with residues in the polybasic patch.

To identify the PIP-binding site and shed light on the mechanism of PIP subtype recognition, we performed extensive MD simulations of the interaction of StarD4 constructs with membranes of various compositions. Both *apo* and cholesterol-bound (*holo*) StarD4 were simulated interacting with membranes of the same composition as the donor vesicles used in the experiments (*i.e.*, a 44:23:23:10 mixture of 1-palmitoyl-2-oleoyl-glycero-3-phosphocholine (POPC), 1-palmitoyl-2-oleoyl-sn-glycero-3-phosphoethanolamine (POPE), cholesterol, with the 10% anionic lipids being either 1-palmitoyl-2-oleoyl-sn-glycero-3-phospho-L-serine (POPS), POPI(4,5)P_2_, or POPI(3,5)P_2_).

**Figure 2:**
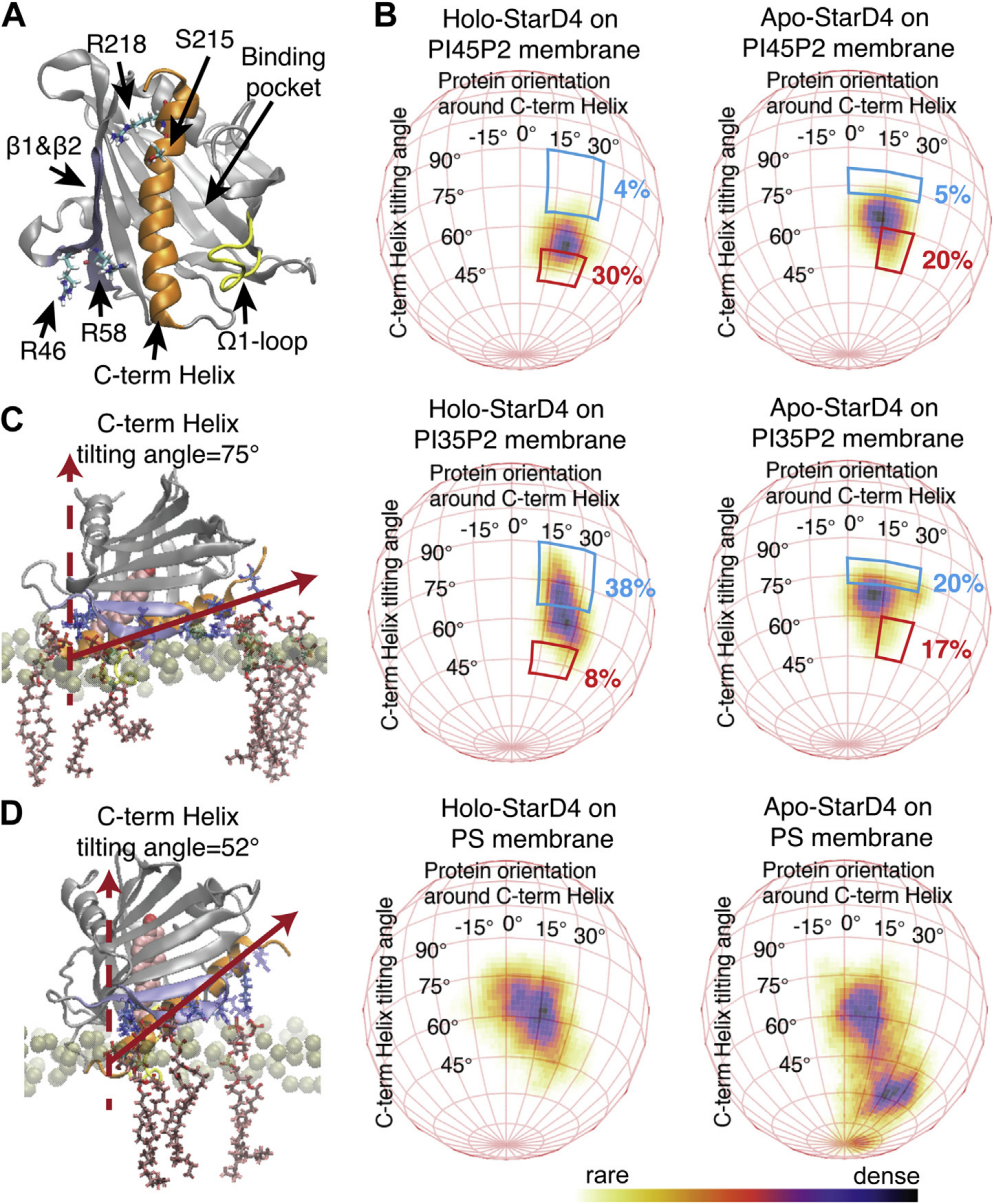
**Representation of the orientation distribution of membrane-embedded StarD4**. *A*, the structure of StarD4 in which the residues R46, R58, S215, and R218 are featured. StarD4 is rendered in *gray* with the C-terminal helix in *orange*, the Ω1-loop in *yellow*, and the β1 and β2 sheets in *blue*. The residues of interest are rendered in “licorice” which draws the atoms as *spheres* and the bonds as *cylinders*. *B*, the probability distribution of the orientation of StarD4 in relation to the lipid bilayers is displayed in a spherical coordinate system with the axes (labeled “C-term Helix tilting angle”, and “protein orientation around the C-term Helix”) defined in Fig. S1. The protein orientations in the regions of the density map enclosed in *blue* and *red* are shown for the holo-StarD4 in panels (*C* and *D*) to illustrate the differences in orientation preferences in the membranes containing PI(3,5)P_2_*versus* PI(4,5)P_2_. StarD4 is rendered in *gray* cartoon with the C-terminal helix in *orange*, the Ω1-loop in *yellow*, and the β1 and β2 sheets in *blue*. The polybasic patch residues are rendered in “licorice” which draws the atoms as *spheres* and the bonds as *cylinders* (*red* and *blue*, respectively). The explicit all-atom lipid membrane model is composed of 400 lipids in symmetric bilayers from a 44:23:23:10 mixture of POPC, POPE, cholesterol, and 10% anionic lipids, that is, either PI(4,5)P_2_, PI(3,5)P_2_, or PS. To simplify the representation, only the phosphate atoms of the upper layer are shown as light-colored *spheres*, with the PIP lipids rendered in “licorice” (see *A* above). The cholesterol ligand is shown in *pink* volume rendering. PIP, phosphatidylinositol phosphate; PS, phosphatidylserine; PI(4,5)P2, phosphatidylinositol 4,5-bisphosphate; PI(3,5)P2, phosphatidylinositol 3,5-bisphosphate; POPC, 1-palmitoyl-2-oleoyl-glycero-3-phosphocholine; POPE, 1-palmitoyl-2-oleoyl-sn-glycero-3-phosphoethanolamine.

The first phase of the simulations sampled the embedding of the StarD4 construct into the membrane in multiple stages for each system (see Experimental procedures). The resulting protein–membrane contact areas were found to involve both the C-terminal helix and the Ω1-loop, while the tail of C-terminal helix and β1&β2 sheets establish sidechain interactions with the headgroups of anionic lipids (Fig. 2*A*) (23). Remarkably, the modes of interaction of the StarD4 protein with the membranes resulted in spatial orientations of the embedded StarD4 that differed for the loading states of the protein (*apo* vs *holo*), and they exhibited clear preferences dependent on membrane composition (Figs. 2*B* and S1). The preferred orientation of holo-StarD4 in PI(3,5)P_2_-containing membrane has the C-term helix lean down more (larger tilting angles) compared to the orientation in PI(4,5)P_2_-containing membranes. Notably, we found that conformations with larger tilting angles have a high probability (∼70%) for binding two or more PIP_2_ lipids in the basic patch at the tail end of the of C-terminal helix (specifically, S215, R218, R219, R222) (Fig. 2*C*). In the orientations with lower tilting angles, the more likely mode (∼60%) is for only one PIP_2_ to be bound at this site (Fig. 2*D*), which is also a preferred binding mode for the PI(4,5)P_2_ lipids. On PS membranes, with less lipid binding overall on the polybasic patch (Table 1), the anchoring of StarD4 in the membrane is weaker, as indicated by the wider sampling of orientations by the protein seen in the corresponding panels of Figure 2*B*. Interestingly, the orientation maps for the protein in the *apo* state exhibit much smaller differences between the different PIP_2_ membrane compositions (although the preference of higher tilting angles for the C-term helix in PI(3,5)P_2_ remains), and they are generally more restricted in the range of angle values.

**Table 1:** The probability (expressed as % occurrence) of finding lipids bound to each basic residue

The anionic lipid is considered to bind to a basic residue if any Oxygen atom of the lipid headgroup is within 4 Å to any Nitrogen or Oxygen atom of the basic residue sidechain. Basic residues chosen for new mutation tests are shown in green. Basic residues for which mutations have been reported in previous studies (Ref. (23)) to hamper the kinetics of StarD4 lipid transfer between PS-containing vesicles are shown in red.

With StarD4 equilibrated in the membrane-embedded states for each of the sampled conditions, we used the MD trajectories to identify the specific modes of interaction of the anionic lipids with the protein likely to underlie the resulting configurations of the protein–membrane interaction modes. Results in Table 1 show the calculated probability of finding one or more anionic lipids bound to a specific basic residue in the polybasic patch. A set of interesting binding patterns emerged from the comparisons of lipid-binding rates in the two loading states of StarD4 (*i.e.*, *apo* vs *holo*):

1. R46 emerged as the primary anionic lipid-binding site for membrane-embedded StarD4, and no significant difference was found between the binding pattern of PI(4,5)P_2_ and PI(3,5)P_2_. Notably, R46 exhibits the highest probability of binding two PIP_2_ lipids at the same time. It is also the highest probability-binding site of PS in membranes containing it as the sole anionic lipid.
2. R58 forms a shared lipid-binding site with R46. Due to the low accessibility of R58 which is spatially occluded in the groove between the C-terminal helix and the Ω1-loop, any lipid that binds R58 is shared with the neighboring R46, which is in the same groove but more accessible. Interestingly, unlike R46, the interaction probabilities with R58 differ for the two PIP_2_ species.
3. Two other residues, S215 and R218, also exhibit different interaction probabilities with the two PIP_2_ species. These differences are also found to be specific to the StarD4-loading state, as they are reversed between *apo—*where S215 prefers PI(3,5)P_2_ and R218 prefers PI(4,5)P_2_—*versus holo* where these preferences are reversed (Table 1).

**Figure 3:**
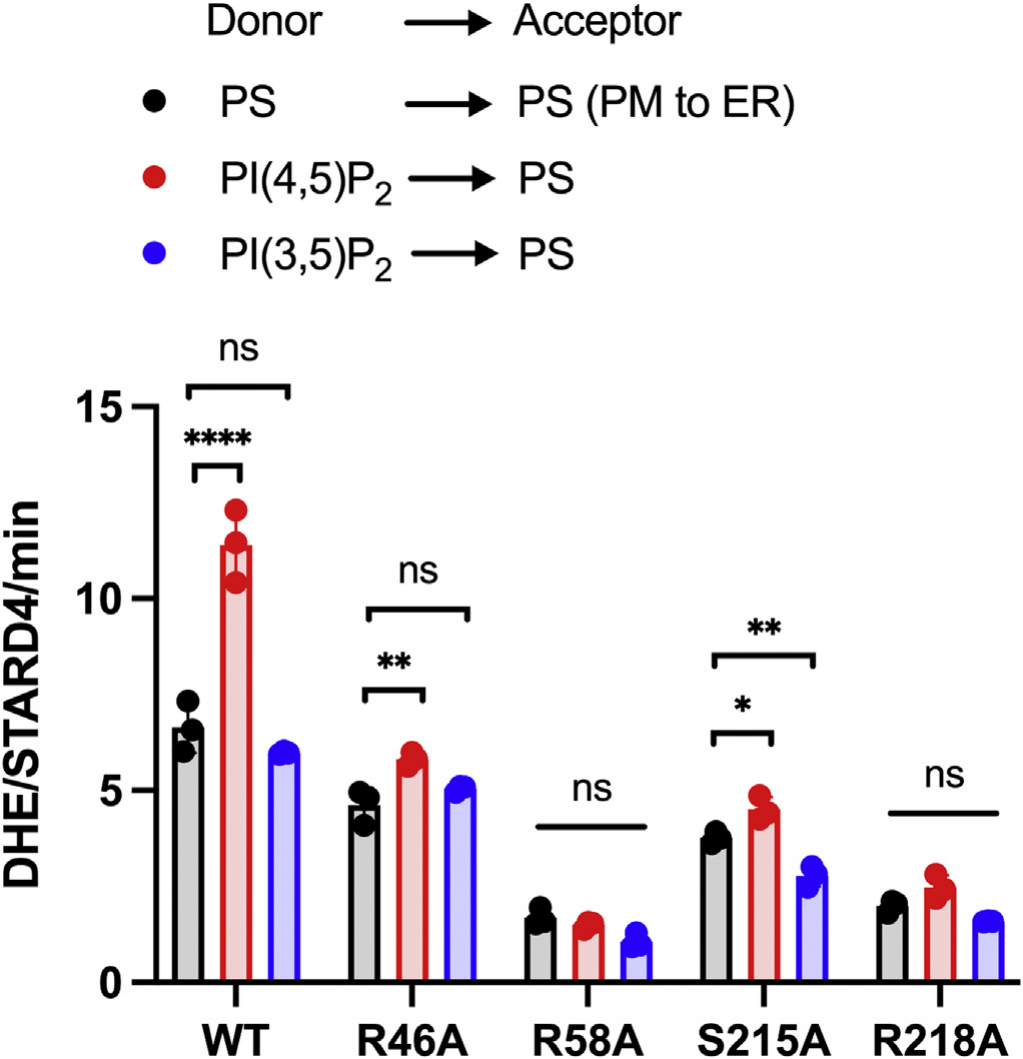
**Mutagenesis of StarD4 PIP-interaction sites**. Quantification of the number of DHE molecules transferred per molecule of WT, R46A, R58A, S215A, or R218A StarD4 per minute using PS-containing acceptor liposomes with PS-containing (*black*), PI(4,5)P_2_-containing (*red*), or PI(3,5)P_2_-containing (*blue*) donor liposomes. Control donor liposomes mimic the cytoplasmic leaflet of the plasma membrane (23 mol % POPS, 31 mol % POPC, 23 mol % POPE, 23 mol % DHE), and control acceptor liposomes mimic ER membranes (5 mol % POPS, 15 mol % liver-PI, 70 mol % POPC, 7 mol % POPE, 3 mol % dansyl-PE). For PIP-containing donor liposomes, 2 mol % POPS was replaced by the same amount of PI(4,5)P_2_ or PI(3,5)P_2_. Experiments were conducted in HK buffer (50 mM Hepes, 120 mM potassium acetate, pH 7.2) at 37 °C. Hundred micromolar donor and hundred micromolar acceptor liposomes were incubated with 1 μM StarD4 added at time zero. Data are plotted from the average of three independent experiments ± SE. ∗*p* < 0.05; ∗∗*p* < 0.01; ∗∗∗∗*p* < 0.0001; ns, non-significant. A summary of statistical significance for this figure is shown in Table S2. DHE, dehydroergosterol; ER, endoplasmic reticulum; PIP, phosphatidylinositol phosphate; PS, phosphatidylserine; PI(4,5)P2, phosphatidylinositol 4,5-bisphosphate; PI, phosphatidylinositol; PI(3,5)P2, phosphatidylinositol 3,5-bisphosphate; dansyl-PE, dansyl-phosphatidylethanolamine; POPC, 1-palmitoyl-2-oleoyl-glycero-3-phosphocholine; POPE, 1-palmitoyl-2-oleoyl-sn-glycero-3-phosphoethanolamine; POPS, 1-palmitoyl-2-oleoyl-sn-glycero-3-phospho-L-serine.

The intriguingly different patterns of the interactions of the four residues with the anionic lipids led to the hypothesis that they would have specific roles in the characteristic effects of PIPs on StarD4 activity and/or the effects of StarD4 on membrane morphology. Guided by these findings, we designed and evaluated several StarD4 mutants in which basic residues in the primary (anchoring) PIP-docking site, R46 and R58, were individually replaced by Ala (Fig. 3). In contrast to Figure 1, the membranes now had 21 to 23 mol % PS. The plasma membrane mimic donor and ER membrane mimic acceptor were also tested as control. These constructs were tested for transport activity using our FRET-based *in vitro* liposome transport assay. Where indicated, 2 mol % PIP_2_ replaced 2 mol % PS in the donor liposomes.

Notable effects were observed in the transport assay in response to inclusion of 2 mol % PI(4,5)P_2_. The sterol transfer activity of WT StarD4 increased by nearly two-fold upon substitution of 2 mol % POPS with the same amount of PI(4,5)P_2_ in donor liposomes. However, substitution of 2 mol % PI(3,5)P_2_ for PS in donor liposomes had no effect on sterol transfer rate. The R46A mutant attenuated sterol transfer activity in PS-containing liposomes by 30% relative to the activity mediated by WT StarD4. In the presence of 2 mol % PI(4,5)P_2_, the attenuation effect of R46A mutant was even higher, with the transfer rate decreased by 50% compared with WT StarD4. However, introducing PI(3,5)P_2_ in donor liposomes did not cause a significant difference in the sterol transfer rate comparing WT and R46A. Notably, the R58A mutant resulted in very strong ablation of the sterol transport activity regardless of the type of anionic lipid added to donor liposomes (Fig. 3 and Table S2*A*). These findings support the key involvement of this site in the PIP-mediated interaction of StarD4 with the membranes.

In addition to the primary PIP-docking site, a secondary site is formed by a cleft between lysines 52 and 219 (23), where residues S215 and R218 are oriented to coordinate the phosphate headgroup of PIPs. Mutation of S215 to alanine reduced StarD4 sterol transfer activity by 40% in PS-containing liposomes, by 53% in PI(3,5)P_2_-containing liposomes and by 60% in PI(4,5)P_2_-containing liposomes. The R218A mutation ablated the sterol transfer activity by more than 70%, with no differences among PS and PIPs (Fig. 3 and Table S2*A*).

We next examined the effects of PIP on sterol transfer activity when added in acceptor liposomes using StarD4 constructs with mutations in the primary or secondary PIP-docking sites. There were at most small differences in transport to acceptor membranes containing PS or either of the PIP_2_s for R46A and S215A StarD4. Similar to the study in donor liposomes, both R58A and R218A mutants ablated more than 80% of the StarD4 sterol transfer activity regardless of whether PS or PIPs was present in the acceptor liposomes (Fig. S2*A* and Table S2).

The experiments described above examined the effects of PIPs when they replace PS solely in donor or acceptor liposomes. Additionally, when PIPs replaced PS in both donor and acceptor liposomes, the overall effect of PIPs on WT StarD4 was dominated by PI(4,5)P_2_ in donor but no significant differences were observed with different PIPs in acceptor. The differences in effects of PIPs or PS on mutant StarD4 activity were not significant (Fig. S2*B* and Table S2).

In addition to the two PIP_2_s, we also examined if three other membrane specific phosphatidylinositol-phosphates, PI(3)P, PI(4)P, and PI(5)P, modulate the StarD4 activity within the synthetic model membranes. PI(3)P was shown to increase sterol transfer rate by WT and S215A StarD4, when added in both donor and acceptor liposomes. PI(4)P decreased transfer in WT donor or acceptor membranes. PI(5)P did not show any effect on StarD4 sterol transfer activity (Fig. S3 and Table S3). Overall, the effects of mutations dominate when we study the sterol transport activity of StarD4 within monophosphorylated PIP-containing liposomes.

### Membrane environment modulates PIP–StarD4 interaction and sterol transfer activity

The difference between the sterol transfer rates produced by StarD4 interactions with membranes enriched in different PIP species suggests a dependence on the specific mode of PIP–StarD4 interaction required to accelerate activity. In a cell, specific PIPs are enriched in certain membrane organelles, but the same PIP can be found in several membranes (33). In the context of sterol transport, the membrane environment of specific PIP species may also contribute to identifying the organelle as either a preferred sterol donor or acceptor to accelerate StarD4 activity.

**Figure 4:**
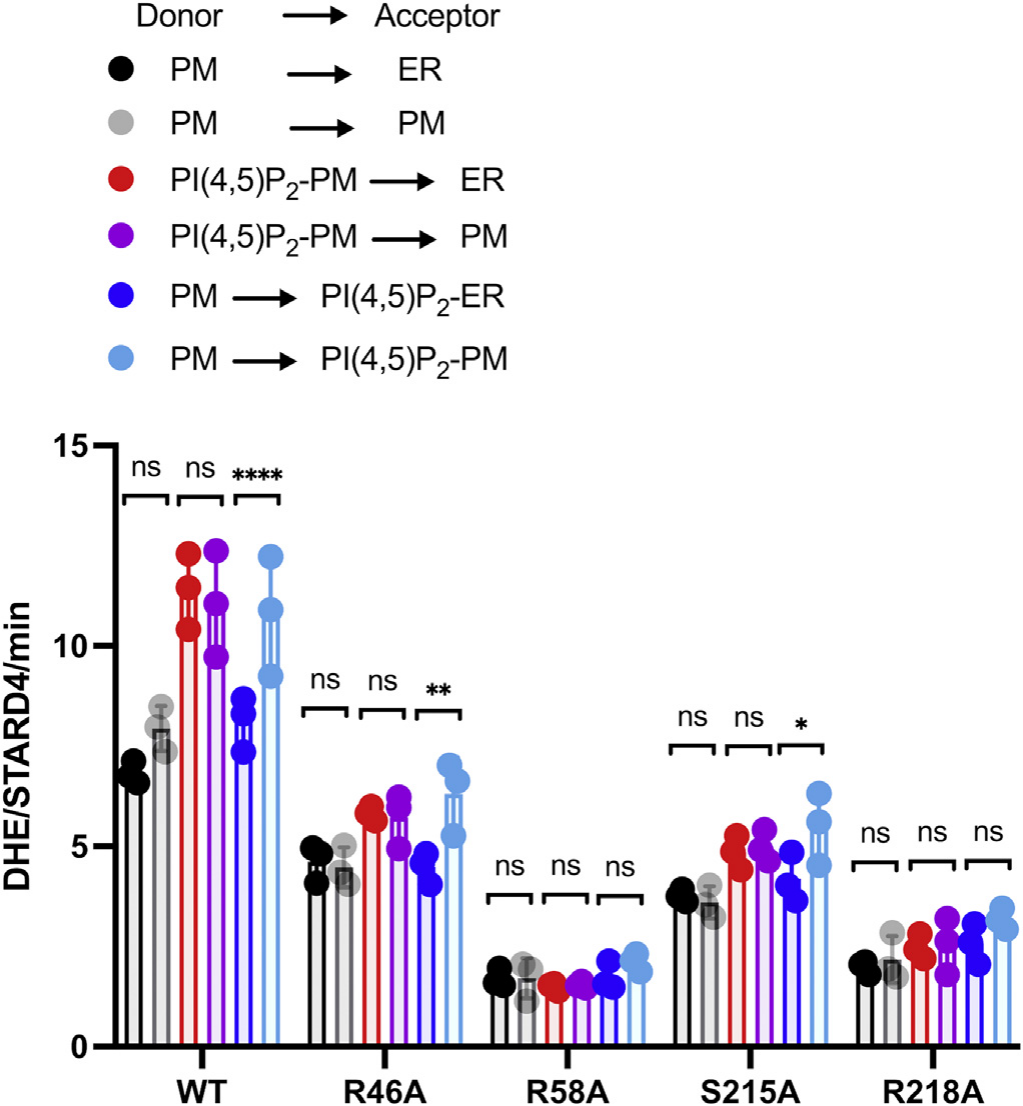
**Membrane environment modulates PIP–Stard4 interactions and sterol transfer activity**. Quantification of the number of DHE molecules transferred per molecule of StarD4 per minute when ER-mimic acceptor liposomes (5 mol % POPS, 15 mol % liver-PI, 70 mol % POPC, 7 mol % POPE, 3 mol % dansyl-PE) were replaced by PM-mimic acceptor liposomes (23 mol % POPS, 31 mol % POPC, 23 mol % cholesterol, 20 mol % POPE, 3 mol % dansyl-PE). For PIP-containing liposomes, 2 mol % anionic lipids (POPS and Liver-PI) were replaced by the same amount of PI(4,5)P_2_. Experiments were conducted in HK buffer (50 mM Hepes, 120 mM potassium acetate, pH 7.2) at 37 °C. Hundred micromolar donor and hundred micromolar acceptor liposomes were incubated with 1 μM WT StarD4 added at time zero. Data are plotted from the average of three independent experiments ± SE. ∗*p* < 0.05; ∗∗*p* < 0.01; ∗∗∗∗*p* < 0.0001; ns, non-significant. A summary of statistical significance for this figure is shown in Table S4. DHE, dehydroergosterol; dansyl-PE, dansyl-phosphatidylethanolamine; ER, endoplasmic reticulum; PM, plasma membrane; PIP, phosphatidylinositol phosphate; PI(4,5)P2, phosphatidylinositol 4,5-bisphosphate; PI, phosphatidylinositol; POPC, 1-palmitoyl-2-oleoyl-glycero-3-phosphocholine; POPE, 1-palmitoyl-2-oleoyl-sn-glycero-3-phosphoethanolamine; POPS, 1-palmitoyl-2-oleoyl-sn-glycero-3-phospho-L-serine.

To test this hypothesis, we performed DHE transport assays in which acceptor liposome compositions mimic the plasma membrane or the ER. In various contexts, 2 mol % PI(4,5)P_2_ was introduced to replace 2 mol % PS in donor or acceptor liposomes. Altering the lipid content of acceptor liposomes without PI(4,5)P_2_ did not alter the sterol transfer activity of WT StarD4 (Fig. 4, black and gray bars). In contrast, the effect of PI(4,5)P_2_ in acceptor liposomes was greater when the PIP was in plasma membrane–like liposomes. (Fig. 4).

We then examined the effects of membrane environment on StarD4 mutants using the same liposomes. R46A and S215A StarD4 showed similar result to WT StarD4, a change in sterol transfer rate was seen only when altering the PIP-containing acceptor environment. R58A and R218A ablated StarD4 sterol transfer activity regardless of which membrane environment was tested. These results indicate that while the presence of membrane-specific PIPs directly affects the StarD4 activity, the overall membrane environment can also contribute to their effects on StarD4 sterol transfer activity (Fig. 4 and Table S4).

### StarD4 induces liposome deformation in specific conditions

**Figure 5:**
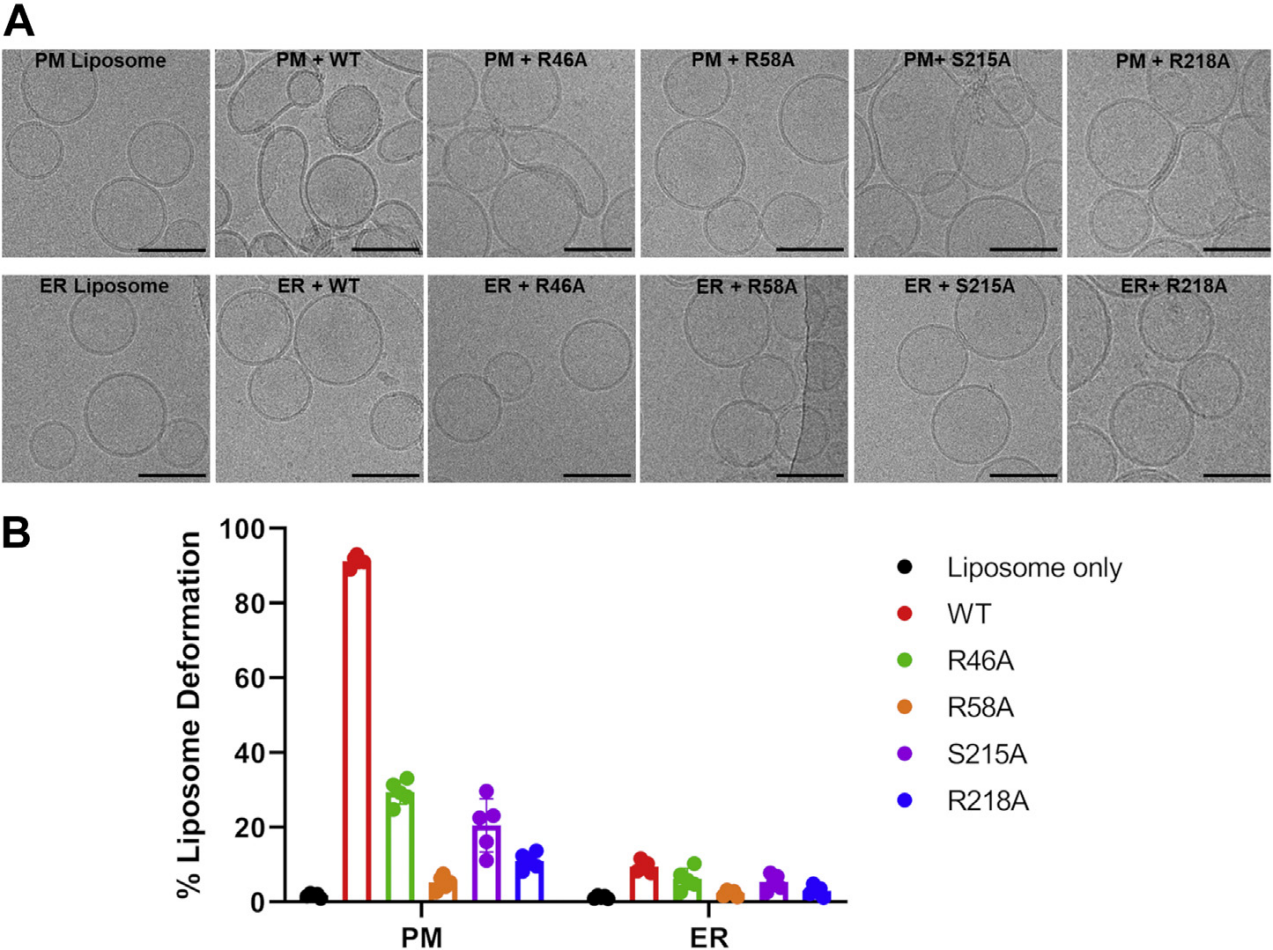
**StarD4 induces liposome deformation under specific conditions**. *A*, cryo-EM images of plasma membrane–mimic liposomes with PI(4,5)P_2_ (2% PI(4,5)P_2_, 21 mol % POPS, 31 mol % POPC, 23 mol % POPE, 23 mol % cholesterol) or ER-mimic liposomes (5 mol % POPS, 15 mol % liver-PI, 70 mol % POPC, 10 mol % POPE) incubated with or without StarD4. Experiments were conducted in HK buffer (50 mM Hepes, 120 mM potassium acetate, pH 7.2) at 37 °C. Four hundred micromolar plasma membrane–mimic or 400 μM ER-mimic liposomes were incubated with 2 μM StarD4 added at time zero. Samples were frozen after 1 h of incubation. The scale bar represents 120 nm. *B*, percentage of deformed liposomes for various conditions in *A*. Percentages (number of deformed liposomes/total number of liposomes) are calculated from the average of five images ± SE, with each image contains from 30 to 50 individual liposomes. ER, endoplasmic reticulum; PI(4,5)P2, phosphatidylinositol 4,5-bisphosphate; PI, phosphatidylinositol; POPC, 1-palmitoyl-2-oleoyl-glycero-3-phosphocholine; POPE, 1-palmitoyl-2-oleoyl-sn-glycero-3-phosphoethanolamine; POPS, 1-palmitoyl-2-oleoyl-sn-glycero-3-phospho-L-serine.

Membrane-associated proteins can alter the morphology of cellular membranes (34). There is evidence that the C-terminal amphipathic helix of StarT-domain proteins can associate with and partially insert into membranes (23, 35), perhaps as part of opening the cholesterol-binding pocket. Transport would require this to be a transient interaction, but under the artificial conditions of our assays, this association might be longer lasting—leading to membrane deformations.

cryo-EM was used to visualize the morphology of plasma membrane–mimic or ER-mimic liposomes in the presence of StarD4. Protein-liposome samples were incubated for 1 h before being frozen rapidly. The morphology of both types of liposomes without added protein was also imaged as control. Both of the control liposomes remained as closed spheres after 1 h incubation in buffer, but some plasma membrane–like liposomes exhibited rupture or nonspherical shapes after incubation with WT, R46A, S215A, or R218A StarD4 (Fig. 5*A*). The percentage of liposomes that were deformed by StarD4 was calculated from 150 to 250 individual liposomes. WT StarD4 induced changes in more than 90% of the plasma membrane–like liposomes. The fraction of deformed liposomes by R46A, S215A, and R218A were reduced compared to WT StarD4 (Fig. 5*B*). No significant morphological change was observed for liposomes in the presence of R58A StarD4. For ER-mimic liposomes, no obvious deformation was observed after mixing with StarD4, regardless of which mutation was added (Fig. 5). These results were consistent with dynamic light scattering (DLS) results (below), suggesting that StarD4 could induce deformation of plasma membrane–like, but not ER-like liposomes under our experimental conditions.

**Figure 6:**
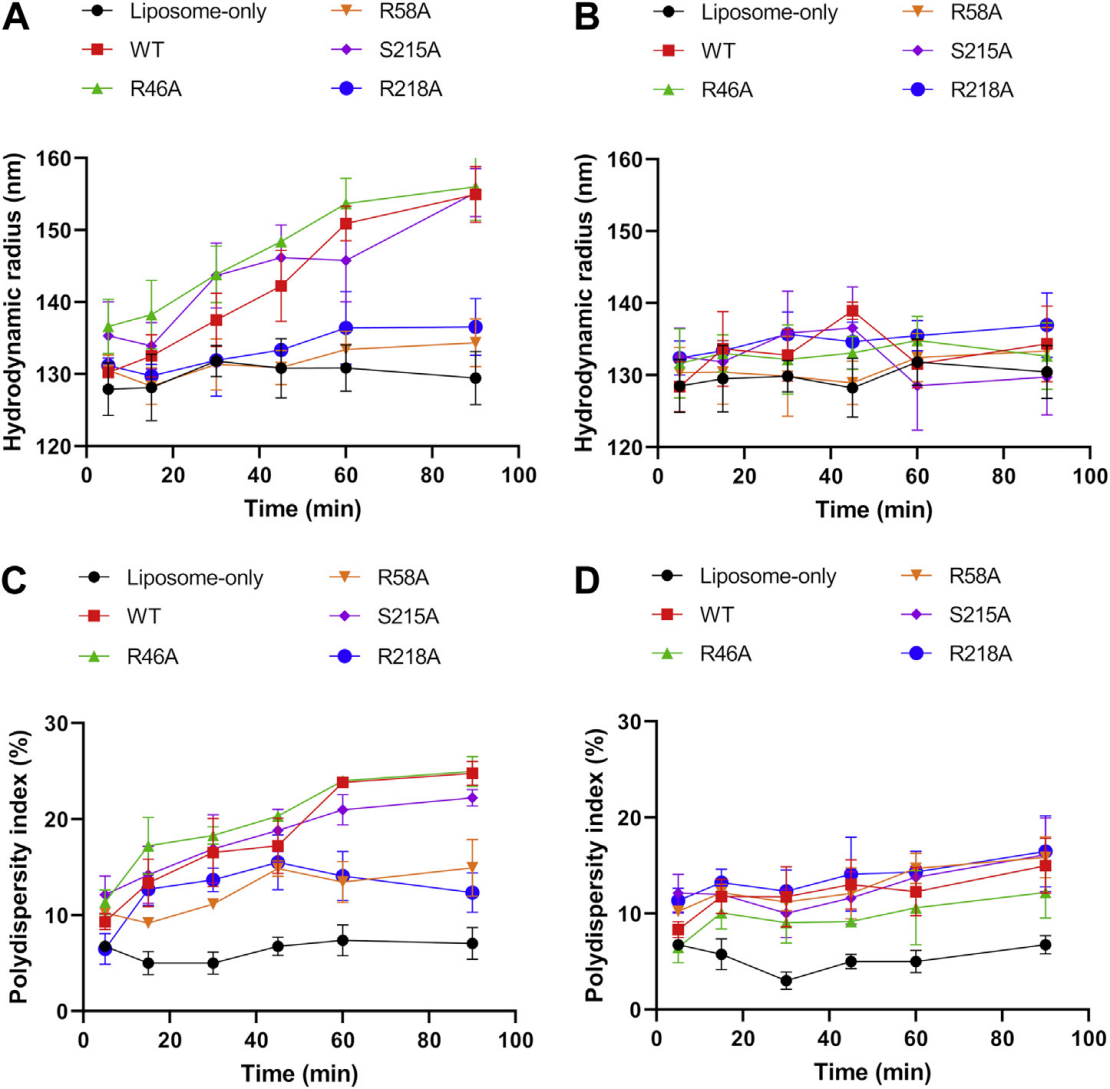
**StarD4 induces liposome deformation under specific conditions**. Hydrodynamic radii and corresponding polydispersity indices of plasma membrane–mimic liposomes (*A* and *C*) and ER-mimic liposomes (*B* and *D*) after incubating with StarD4 by dynamic light scattering. Plasma membrane–mimic liposomes contain 2% PI(4,5)P_2_, 21 mol % POPS, 31 mol % POPC, 23 mol % POPE, 23 mol % cholesterol, and ER-mimic liposomes contain 5 mol % POPS, 15 mol % liver-PI, 70 mol % POPC, 10 mol % POPE. Experiments were conducted in HK buffer (50 mM Hepes, 120 mM potassium acetate, pH 7.2) at 37 °C. Two hundred micromolar donor or two hundred micromolar acceptor liposomes were incubated with 1 μM StarD4 added at time zero. Data are plotted from the average of three independent experiments ± SE. ER, endoplasmic reticulum; PI(4,5)P2, phosphatidylinositol 4,5-bisphosphate; PI, phosphatidylinositol; POPC, 1-palmitoyl-2-oleoyl-glycero-3-phosphocholine; POPE, 1-palmitoyl-2-oleoyl-sn-glycero-3-phosphoethanolamine; POPS, 1-palmitoyl-2-oleoyl-sn-glycero-3-phospho-L-serine.

We used DLS to characterize the change of liposome effective diameter upon adding protein. StarD4 was incubated with plasma membrane–like or ER-like liposomes. Both the hydrodynamic radius and the corresponding polydispersity index of liposomes were recorded for 90 min. As shown previously, the cholesterol transfer process equilibrates in less than 5 min for WT and somewhat more slowly for mutants (7, 23). Both the hydrodynamic radius and the polydispersity index of plasma membrane liposomes increased upon adding WT, R46A, and S215A StarD4, which mean that there were changes in the effective diameter or shape of liposomes under these conditions (Fig. 6, *A* and *C*). However, R58A and R218 StarD4 did not change the diameter of the liposomes as much upon interaction (Fig. 6, *A* and *C*). For ER-mimic liposomes, we did not detect large changes in either hydrodynamic radius or the polydispersity index up to 90 min of protein interactions (Fig. 6, *B* and *D*).

To exclude the possibility of liposome fusion, we conducted another experiment in which liposomes labeled with 0.5 mol % 1,2-dioleoyl-sn-glycero-3-phosphoethanolamine-N-(7-nitro-2–1,3-benzoxadiazol-4-yl) (NBD-DOPE) and 1 mol % 1,2-dioleoyl-sn-glycero-3-phosphoethanolamine-N-(lissamine rhodamine B sulfonyl) (Rho-DOPE) were mixed with unlabeled liposomes. Fusion of liposomes would result in dilution of the FRET donor and acceptor pair in liposomes, which could be detected by an increase in NBD fluorescence. We did not detect any NBD fluorescence increase in plasma membrane–mimic or ER-mimic liposomes after adding StarD4 and incubating for 15 min, which was the time when most sterol transfer took place. As a positive control, 50 mM CaCl_2_ was also added to induce liposome fusion, resulting in an increase of NBD fluorescence in both cases (Fig. S4). This suggested that StarD4 did not induce liposome fusion under our experimental conditions.

The initial protein–membrane interaction has been evaluated through binding between tryptophan in StarD4 and sterol in membranes. Initial binding between protein and membranes are tighter for StarD4 proteins that induce membrane deformation than for mutants that do not induce membrane deformation, regardless of whether PS or PIPs was presented in membranes (Fig. S5).

## Discussion

The mechanism(s) by which cells generate and maintain different sterol concentrations in various organelles remains an important, open question in cell biology (3). One mechanism would depend on the composition of other lipids in the membranes, with cholesterol enriched in membranes that stabilize cholesterol based on the properties of headgroups and acyl chains (10, 11, 36). The plasma membrane, with high levels of sphingolipids and phosphatidylcholine as well as low levels of unsaturated acyl chains, is a membrane that stabilizes cholesterol well (27). In contrast, the ER, which has more lipid unsaturation and more lipids with small headgroups, would provide weak stabilization (10, 27, 37). These differences in lipid composition could contribute to the differences in sterol content among organelles, which could be maintained even if the membranes were at equilibrium. If sterol transport proteins simply accelerated the movement of sterol between membranes with no selectivity, the plasma membrane would remain enriched in sterols relative to the ER. Indeed, when we injected β-cyclodextrin into the cytoplasm of cells, the rate of transport between the plasma membrane and the ERC became faster, but there was little change in the distribution of DHE among organelles (7, 38).

In an alternative model, some sterol transport proteins would be able to move sterol selectively from one organelle to another. For example, OSBP (30) and Osh4P (31) can exchange cholesterol for PI(4)P, and this would allow cholesterol to be enriched in the PI(4)P-enriched membrane by using the free energy provided by the PI(4)P concentration ratio, a process that can be maintained by hydrolysis of the PI(4)P in the donor organelle (30, 31). Cholesterol can move against its concentration gradient from the ER to the Golgi by this mechanism (30), but it is unclear what fraction of sterol transport from ER to Golgi is mediated by this mechanism.

StarD4 is an important transporter of sterol between membranes including the plasma membrane and the ERC (6, 7, 38). Increased levels of StarD4 are also associated with increased sterol esterification by ACAT and inhibition of SREBP-2 processing, indicating that StarD4 accelerates delivery of cholesterol to the ER (7, 18). It has not been clear if these increases in transport rate are simply due to increased rates of exchange or if StarD4 can selectively transport lipids from one organelle to another. Data presented in this article show that StarD4 can transport sterol among any membranes that contain anionic phospholipids, but the rates of transport are affected significantly by the specific PIP content of the donor and acceptor organelles.

We demonstrate a PIP_2_-selective mechanism by which StarD4 can preferentially extract sterol from membranes containing certain PIPs (especially PI(4,5)P_2_), with less effect when the same PIPs are in the acceptor membranes or when other anionic lipids, including monophosphorylated PIPs, are in the liposomes. This is shown by experiments in which donor and acceptor liposomes were mixed with StarD4, and various PIPS were utilized to substitute for PS. This indicates that the modulation is unique to the choice of PIPs.

Together, the results from the computational modeling of the systems and the MD simulations support the mutagenesis results and point to the mechanisms underlying the experimental findings. Specifically, (i)-the clear dependence of the orientation toward the membrane on the PIP_2_ composition that we established for loaded StarD4 and (ii)-the differential mode of binding to the (S215, R218, R219, R222) cluster of residues suggest how the StarD4–membrane interaction mode is established as a factor in the PIP_2_ regulation of StarD4 kinetics.

As discussed in Results, R58 is spatially quite occluded in the groove between the C-terminal helix and the Ω1-loop. Thus, any molecule that binds to R58 is necessarily shared with the neighboring R46, which is in the same groove but more accessible. Thus, total lipid binding to R46 includes binding to R58. Clearly, the tight environment of R58 in the WT structure will change much when it is replaced by an Ala in R58A. The effect of the structural perturbation is clearly recognizable in Figure 3 by the drastic and nonselective reduction in the number of DHE transferred, compared to the results for all other WT and mutant constructs. The overall mechanism by which cholesterol enters and leaves StarD4 remains uncertain. A previous study indicated that the amphipathic C-terminal helix may insert into the lipid bilayer to some extent (23). The main role of R46 and R58 is to selectively bind anionic phospholipids.

The PIP specificity of StarD4 may play an important role in intracellular sterol transport. It has been noted that increased StarD4 expression leads to increased cholesterol esterification in the ER by ACAT (7). One report shows that the maturation and fusion of autophagosomes, which contain PI(3)P (39, 40) require an increase in cholesterol levels, which is sensed by ORP1L (41). PI(3,5)P_2_ is enriched in late endosomes, and it has been proposed that cholesterol in late endosomes can affect mTOR signaling (42). We propose that StarD4 can play a prominent role in distributing cholesterol from the PI(4,5)P_2_-rich plasma membrane, which is the largest cellular pool of cholesterol, to other membranes, which are good sterol acceptors from StarD4.

In a previous article, StarD4 was shown to modulate overall cellular lipid composition and influence the biophysical properties of the plasma membrane (29). StarD4 mediates cholesterol transport between membranes in distinct steps. First, apo-StarD4 removes sterol from donor membranes to form a StarD4-sterol complex. This complex then diffuses from a donor to an acceptor membrane. Lastly, StarD4 releases the sterol into the acceptor membrane.

We previously reported that NBD fluorophore attached to a sidechain facing the core of StarD4 in the C-terminal amphipathic helix inserts into the bilayer of membranes (23). For transport, this insertion needs to be very transient to allow release of StarD4 from the membrane. Upon longer incubations, we observed that StarD4 can deform membranes—especially membranes with compositions favorable to sterol extraction. This may reflect that a small percentage of StarD4 interactions are slowly reversible or irreversible in our model system.

To summarize, this work presents a systematic study of the modulations of varying PIPs on cholesterol transfer rate by StarD4 and the effects of StarD4 on membrane morphology. We show that StarD4 recognizes membrane-specific PIPs through specific interaction with the geometry of the PIP headgroup as well as the surrounding membrane environment. A subset of basic residues is involved in specific modes of interaction with different PIPs and efficient sterol transport between PIP-containing membranes. The recruitment of apo-StarD4 to PI(4,5)P_2_-containing membrane would accelerate sterol binding and formation of a StarD4-sterol complex. When the StarD4 dissociation from the PI(4,5)P_2_ membrane is not complete, this may result in membrane deformation. Future work could examine how these differences in approach and binding of StarD4 to membranes relate to the clear preference to extract sterol from PI(4,5)P_2_ membranes and to release it to acceptor membranes.

## Experimental procedures

### Materials

ergosta-5,7,9(11),22-tetraen-3ß-ol, cholesterol, and calcium chloride (CaCl_2_) were purchased from Sigma. POPC, POPE, POPS, L-α-PI (Liver PI), 1,2-dioleoyl-sn-glycero-3-phosphoethanolamine-N-(5-dimethylamino-1-naphthalenesulfonyl) (dansyl-PE), 1-stearoyl-2-arachidonoyl-sn-glycero-3-phospho-(1′-myo-inositol-4′,5′-bisphosphate) (PI(4,5)P_2_), 1-stearoyl-2-arachidonoyl-sn-glycero-3-phospho-(1′-myo-inositol-3′,5′-bisphosphate) (PI(3,5)P_2_), 1,2-dioleoyl-sn-glycero-3-phospho-(1′-myo-inositol-3′-phosphate) (PI(3)P), 1,2-dioleoyl-sn-glycero-3-phospho-(1′-myo-inositol-4′-phosphate) (PI(4)P), 1,2-dioleoyl-sn-glycero-3-phospho-(1′-myo-inositol-5′-phosphate) (PI(5)P), NBD-DOPE, and Rho-DOPE were purchased from Avanti Polar Lipids (Alabaster). The concentrations of unlabeled lipids were determined by dry weight and that of fluorescent lipids by absorbance using εNBD-DOPE = 21, 000 M^−1^ cm^−1^ at 460 nm in methanol and εRho-DOPE = 95,000 M^−1^ cm^−1^ at 560 nm in methanol.

### WT and mutant mStarD4 constructs

The cDNA encoding WT StarD4 was subcloned into the pET-SUMO vector (Invitrogen) (7, 23, 43). R46A, R58A, S215A, and R218A mStarD4 were generated using site-directed mutagenesis. These StarD4 constructs were expressed in *Escherichia coli* BL21(DE3) cells and purified as described previously (7, 23). Purified protein was stored at −80 °C.

### Liposomes

Lipids were prepared as previously described (7, 44). Liposomes referred to as “donors” or “acceptors” were used in a sterol-transfer assay. For FRET experiments, the base composition of donor liposomes was 31 mol % POPC, 23 mol % POPE, 23 mol % POPS, 23 mol % DHE, and the acceptor liposomes were 70 mol % POPC, 7 mol % POPE, 15 mol % liver PI, 5 mol % POPS, 3 mol % dansyl-PE. Modified lipid compositions were used as described in the text. For DLS and cryo-EM experiments, donor liposomes contained 2% PI(4,5)P_2_, 21 mol % POPS, 31 mol % POPC, 23 mol % POPE, 23 mol % cholesterol and acceptor liposomes contained 5 mol % POPS, 15 mol % liver-PI, 70 mol % POPC and 10 mol % POPE.

### Sterol transfer assay

The sterol transport activity of StarD4 was measured by a FRET assay, as previously described (7, 23). Experiments were performed in quartz cuvettes (100 μl) in HK buffer (50 mM Hepes, 120 mM potassium acetate, pH 7.2) equilibrated at 37 °C on a SpectraMax M3 fluorometer (Molecular Devices). FRET traces were fit by a single exponential. Data represent averages (±SEM) of at least three independent experiments.

### Dynamic light scattering

DLS experiments were performed on an Anton Paar Litesizer 500. Experiments were conducted in HK buffer at 37 °C. Two hundred micromolar donor or two hundred micromolar acceptor liposomes were incubated with 1 μM StarD4 from time zero. The hydrodynamic radius and corresponding polydispersity index were measured. Data represent averages (±SEM) of three independent experiments.

### FRET measurements of liposome fusion

FRET experiment was done using a donor-acceptor pair of NBD-DOPE/Rho-DOPE. NBD fluorescence was measured with an excitation wavelength of 465 nm and an emission wavelength of 534 nm for 15 min, when most of the sterol transfer happened. For FRET in labeled vesicles, F samples had a mixture of unlabeled lipid, lipid labeled with 0.5 mol % NBD-DOPE and 1 mol % Rhod-DOPE. Fo samples contained a mixture of unlabeled lipid and lipid labeled with 0.5 mol % NBD. Background for F samples had unlabeled lipid with same amount of acceptor as in the F samples. Background samples for Fo contained pure unlabeled lipid. For FRET experiments, liposomes were incubated in the presence and absence of 1 μM StarD4 or in the presence of 50 mM CaCl_2_ as a positive control.

### Cryogenic transmission electron microscopy

Samples were incubated for 1 h before loading to the EM grids. Sample freezing was done using a Vitrobot Mark IV plunge freezer (ThermoFisher). Holey carbon film on 400 mesh copper grids (Quantifoil R1.2/1.3) were plasma cleaned (Pelco EasiGlow) before applying sample solution. Sample was added, first manually blotted with blotting paper, and then the sample was loaded a second time, incubated for 60 s on the grid and blotted automatically for 2.5s and blot force 1, prior to vitrification by plunge freezing into liquid ethane chilled with liquid nitrogen (45). Grids were imaged using a 200-kV Glacios microscope (ThermoFisher) in a Weill Cornell Medical College Core Facility. Data was automatically collected, using the SerialEM, at a nominal magnification of 8,500X (pixel size of 2.5 Å). The total electron dose for the K2 direct electron detector (Gatan) was set to 20 e/Å^2^, fractionated over 80 frames before aligning and adding frames using IMOD software package software (46, 47).

### FRET measurements of initial protein–membrane binding

A FRET donor and acceptor pair, tyrosine (in StarD4) and DHE (in membranes), was used as described previously (48). Tyrosine fluorescence was measured using a fluorescence spectrophotometer with an excitation wavelength of 270 nm and emission wavelength of 305 nm. F samples contained StarD4 and liposomes with unlabeled lipid and 23 mol % DHE, while Fo samples contained StarD4 and liposomes with unlabeled lipid and 23 mol % cholesterol in place of DHE. Background samples contained unlabeled lipid without peptide. For FRET experiments, plasma membrane–like liposomes were titrated into 2 μM StarD4/HK buffer at concentrations ranging from 0 to 1000 μM lipid. Backgrounds were liposomes injected into HK buffer. Fluorescence was read after titrating in liposomes and incubating for 1 min. Data represents averages (±SEM) of three independent experiments.

### Computational procedures

#### Preparation of molecular systems

##### Models of StarD4

The structure of *apo*-StarD4 was taken from the X-ray structure (PDB ID: 1jss) where residues 24 to 222 are resolved (22). K223 and A224 are added using Modeller 9.23 software, resulting in the conformation of StarD4 22-224 segment with acetylation on the N-terminus and carboxylation on the C-terminus (49). The initial structure of cholesterol bound (*holo*) StarD4 was obtained by docking cholesterol in the hydrophobic pocket of StarD4 using Schrodinger Induced Fit Docking protocol (50, 51, 52). *Apo-* and *holo-*StarD4 structures are solvated in 0.15 M K^+^Cl^−^ ionic aqueous solution and equilibrated using the NAMD simulation platform version 2.12 (53).

##### Models of the lipid membrane

An explicit all-atom lipid membrane model was built from 400 lipids in symmetric bilayers composed of a 44:23:23:10 mixture of POPC, POPE, cholesterol, and 10% anionic lipids, that is, either POPS, POPI(4,5)P_2_, or POPI(3,5)P_2_. The initial bilayer structure was assembled on the CHARMM-GUI webserver (54).

#### Preparation protocol for StarD4–membrane interactions and embedding simulations

1. Equilibration of StarD4 in aqueous solution.
2. Equilibration of the anionic lipids in the membrane in the field of an approaching StarD4 molecule.
3. Simulation of the StarD4–membrane interaction and its embedding in the membrane.

##### Equilibration of StarD4 in aqueous solution

To study the conformational changes of StarD4 before membrane interaction is established, *apo-* and *holo-*StarD4 were simulated in aqueous environments using OpenMM software. Twelve replicas of systems obtained from the NAMD equilibration were run for 1 μs/each simulation. The OpenMM simulations in NPT ensemble (T = 310K, *p* = 1 atm) use a 4 fs integration time-step and a Monte Carlo Membrane Barostat. Following clustering analysis, three representative conformations for *apo-* and for *holo-*StarD4, respectively, were used to build the atomistic models for the simulations of StarD4–membrane complexes.

##### Equilibration of the anionic lipids in the membrane in the field of an approaching StarD4 molecule

To evaluate the effects of long-range electrostatic interactions between StarD4 and membrane, we employed the mean-field model approach to assess the orientation of StarD4 in approaching the negatively charged membranes and the corresponding rearrangement of anionic lipids of the membrane in response to the approaching StarD4 (55, 56, 57, 58). MFM sampling was shown to accelerate the exploration under important degrees of freedom (electrostatics, lipid mixing) for the long-range interaction to establish an optimized state for the StarD4–membrane combination.

Briefly, StarD4 is treated at the detailed 3D atomic level, while the membrane is considered as two-dimensional smooth charged surface representing the lipid polar headgroups. StarD4 positions are probed facing the membrane from different orientations, with all atoms at least 2 Å away from the membrane. The rearrangement of membrane lipid position corresponding to the multiple StarD4 poses are carried out by self-consistent minimization of the governing mean-field–based free energy function (F) that is given as a sum of electrostatic ($F_{el}$), and translational entropy of mobile ions ($F_{IM}$) depends on the local lipid component densities $\varphi_{\left(x,y\right)}$ and lipid mixing entropy ($F_{lip}$) depends on the mobile ion concentrations $c^{-}$ and $c^{+}$ (55, 58). The simulation was carried out in a (256 Å)^3^ cubic space, with 0.15 M ionic solution of monovalent counterions (corresponding to λ = 8.09 Å Debye length) under the temperature of 310K. The best orientation of membrane approaching StarD4 is selected as the position that results in the strongest absorption energy.

The combined models of StarD4–membrane complexes described below were built with optimized lipid arrangement and protein orientations obtained from this procedure. To this end, the best orientation of the membrane approaching StarD4 is selected as the position that results in the strongest absorption energy, and the positions of anionic lipids around of protein in the atomistic membrane model are assigned according to the steady state lipid density map predicted by the MFM calculation.

##### Simulation of the StarD4–membrane interaction and its embedding in the membrane

For the simulation of the STARD4–membrane complexes, the starting model was constructed by positioning the protein so that the minimum distance between any protein atoms to the membrane anionic lipid head surface was larger than 2 Å. The systems were solvated using 0.15 M K^+^Cl^-^ ionic solution, resulting in a system size of ∼138,000 atoms. Each StarD4–membrane complex was subjected to a multistep equilibration protocol: restraining was applied on the protein backbone atoms, the heavy atoms of the docked cholesterol, and the heavy atoms of the membrane lipids heads, with the force constant gradually decreasing from 1 $\text{kcal}/\text{mol}\cdot {Å}^{2}$ to 0.1 $\text{kcal}/\text{mol}\cdot {Å}^{2}$ in three 0.5 ns steps. The equilibration step was carried out in NAMD at temperature of 310K in NPT ensemble as previously established (59, 60).

The final frames of the previous phase were used as an input to run MD simulations using the OpenMM software (61). Each loading state (with either apo- or holo-StarD4 interacting with membrane containing 10% anionic lipid (either PI(4,5)P_2_, or PI(3,5)P_2,_ or PS) was simulated with nine replicas for ∼5.5 μs/each for a total of ∼50 μs. The simulation in OpenMM is carried out using four fs integration time-step, in NPT ensemble (T = 310K, *p* = 1 atm) using Monte Carlo Membrane Barostat, with isotropic XY ratio.

In the first 500 ns of the simulation, the relative position of StarD4 (z-distance to the membrane surface) and the orientation of StarD4 (z-distance between the left-side-half and right-side-half of StarD4 and between the top-side-half and the bottom-side-half the StarD4) was strongly constrained (20 $\text{kcal}/\text{mol}\cdot {Å}^{2}$), allowing the equilibration of the membrane lipid rearrangement with StarD4 being adjacent but not closer. Then, all the constrains are released, so StarD4 is allowed to establish direct contact with the membrane, and the simulation was carried out for another ∼5 μs/each.

Membrane embedding of StarD4 is achieved when the contact surface area between the ω1-loop and the membrane is >200 ${Å}^{2}$, and the contact surface area between the β9 & C-term-helix and the membrane is >150 ${Å}^{2}$. After the 5.5 μs simulation, multiple (2, 3, 4) trajectories were found with the StarD4 embedded into the membrane for each loading state. Each membrane-embedded trajectory was used to generate 12 trajectories per loading state, and each was simulated for another 2.2 μs for a total of 26.4 μs/each to obtain the sample of membrane-embedded StarD4 for each loading state. The last 720 ns from each trajectory was used for the analysis described in the text.

## Data availability

All experimental data are contained in the article. The corresponding author (F. R. M) can be contacted for additional experimental information. Computational data used to arrive at the conclusions presented in the article are available upon reasonable request from (H. W.). For the molecular constructs used in computational experiments, we utilized Modeller version 9v1 and VMD version 1.9.1. Atomistic MD simulations were carried out with OpenMM 7.5. Computational analysis was carried out using a combination of python scripts and in-house scripts available on GitHub https://github.com/weinsteinlab/

## Supporting information

This article contains supporting information.

## Conflict of interest

The authors declare that they have no conflicts of interest with the contents of this article.
