# Supplementary material for: Phosphatidylinositol phosphates modulate interactions between the StarD4 sterol trafficking protein and lipid membranes
Source: J Biol Chem. 2022 May 20;298(7):102058. doi: 10.1016/j.jbc.2022.102058 (PMC9207681; doi:10.1016/j.jbc.2022.102058)
Supplement: Supporting information [file mmc1.pdf]

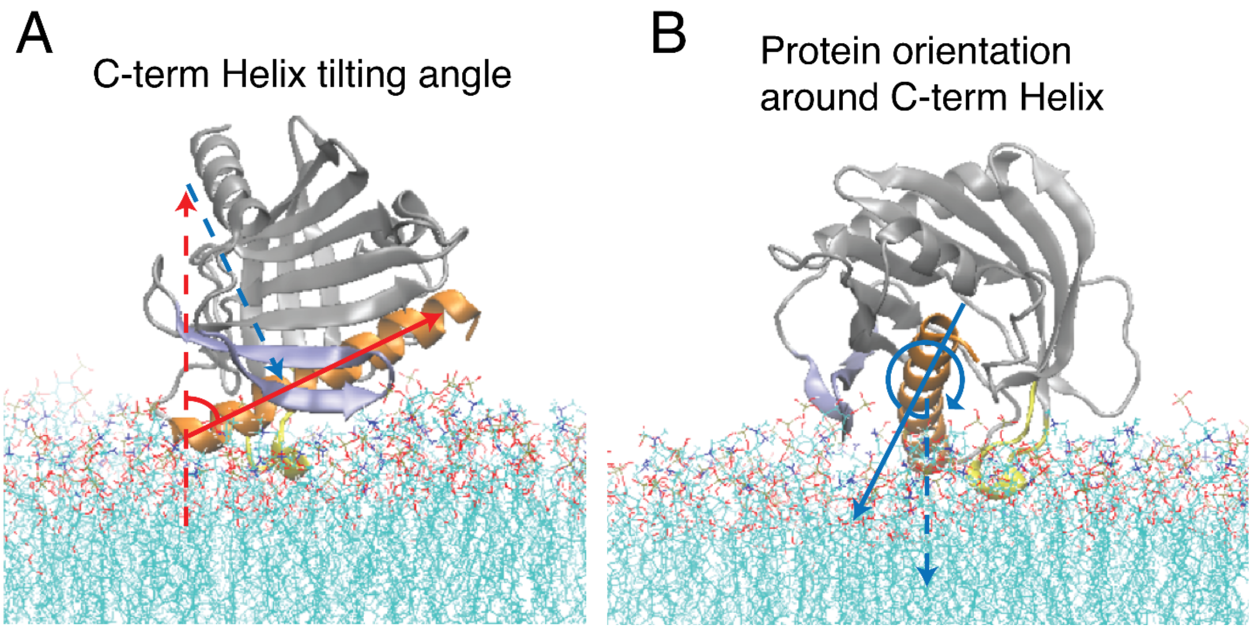

Supporting Figure 1: Definitions of the orientations of membrane embedded StarD4 observed in the MD simulations. The preferred positioning of the StarD4 protein when it is embedded in membranes with different lipid compositions is defined by two angles of the spherical coordinate system termed **(A) C-term Helix tilting angle** and **(B) Protein orientation around C-term Helix**.

(A) C-term Helix tilting angle: The "axis of the C-term Helix" (red line) is defined as the first principal component of the Cα atoms of the C-term Helix. The "axis of membrane norm" is the dashed red line in Panel A. With these definitions, the "**C-term Helix tilting angle**" measures the angle between the "axis of the C-term Helix" and the "axis of membrane norm". The dashed blue line is the orthogonalized vector of membrane norm that is perpendicular to the axis of the C-term Helix, and is termed "reference orientation that faces the membrane".

(B) The orientation of the protein around the C-term Helix: The "axis of protein around C-term Helix" (blue line) is a perpendicular to the "axis of the C-term Helix" that passes through the center of mass of the protein Cα atoms. The "reference orientation that faces the membrane" (dashed blue line in both A and B) is the orthogonalized vector of membrane norm that is perpendicular to the "axis of the C-term Helix", obtained by vector subtraction of the membrane norm and its projection on the "axis of the C-term Helix". With these definitions, the "**protein orientation around C-term Helix**" measures the angle between the "axis of protein around C-

term Helix” and the “reference orientation that faces the membrane”; the clockwise direction is assigned positive values, and the anti-clockwise direction is negative.

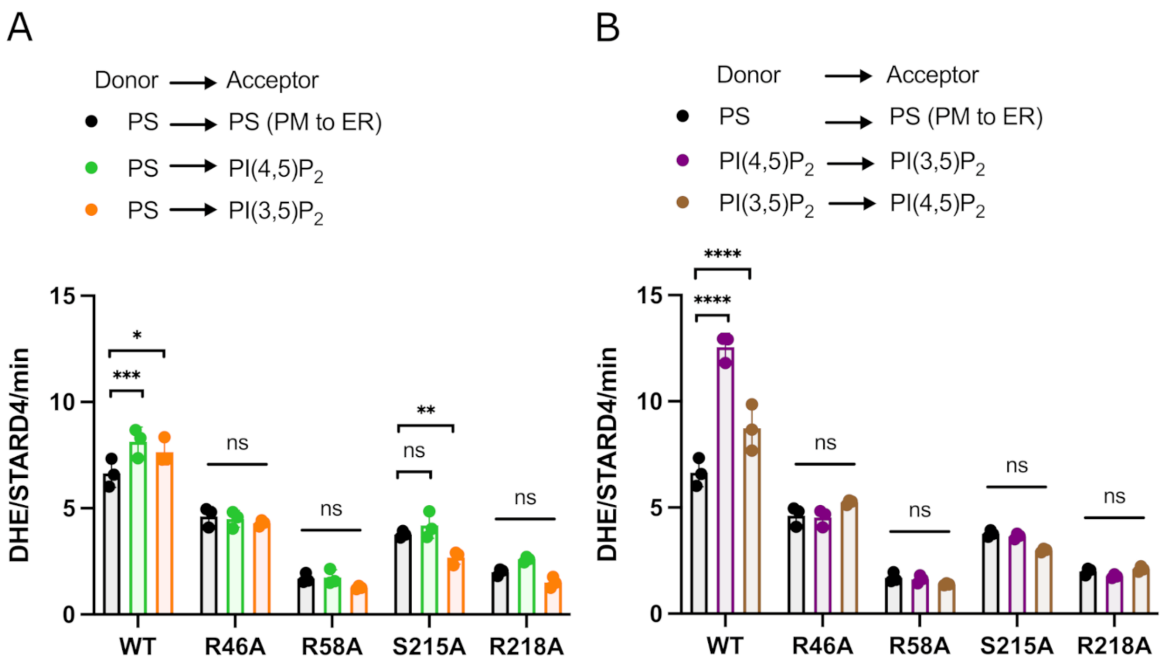

Supporting Figure 2. Mutagenesis of StarD4 PIP interaction site. Quantification of the number of DHE molecules transferred per molecule of WT, R46A, R58A, S215A or R218A StarD4 per minute using **(A)** PS-containing donor liposomes with PS-containing (black), PI(4,5)P2-containing (green), or PI(3,5)P_2-containing (orange) acceptor liposomes; **(B)** PI(4,5)P2-containing donor liposomes with PI(3,5)P_2-containing acceptor liposomes (purple), or PI(3,5)P2-containing donor liposomes with PI(4,5)P_2-containing acceptor liposomes (brown). Control donor liposome mimics cytoplasmic leaflet of the plasma membrane (23 mol% POPS, 31 mol% POPC, 23 mol% POPE, 23 mol% DHE), and control acceptor liposome mimics ER membrane (5 mol% POPS, 15 mol% liver-PI, 70 mol% POPC, 7 mol% POPE, 3 mol% Dansyl-PE). For PIP-containing liposomes, 2 mol% anionic lipids (POPS) were replaced by the same amount of PI(4,5)P_2 or PI(3,5)P_2. Experiments were conducted in HK buffer (50 mM HEPES, 120 mM potassium acetate, pH 7.2) at 37 °C. 100 µM donor and 100 µM acceptor liposomes were incubated with 1 µM StarD4 from time zero. Data are plotted from the average of three independent experiments ± SE. *, $p < 0.05$; **, $p < 0.01$; ***, $p < 0.001$; ****, $p < 0.0001$; ns, non-significant. Detailed statistical analysis is in Supporting Table 2.

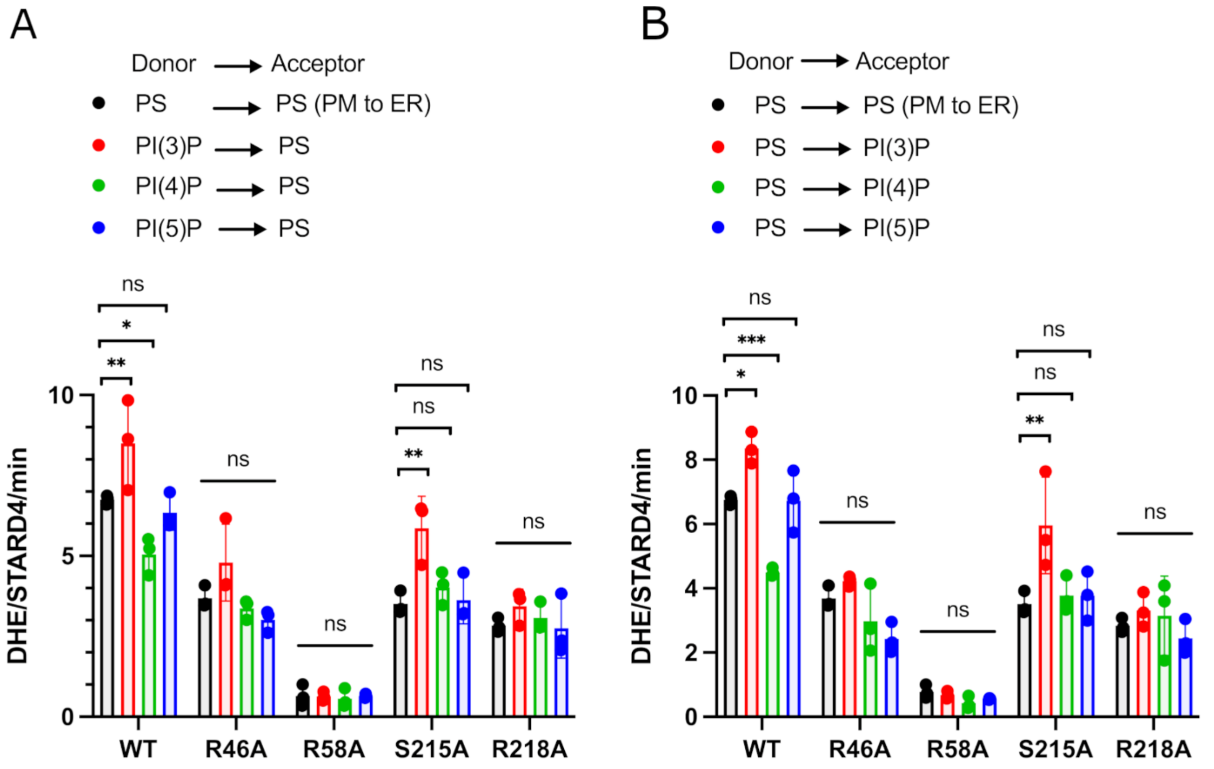

Supporting Figure 3. Quantification of the number of DHE molecules transferred per molecule of WT, R46A, R58A, S215A or R218A StarD4 per minute using **(A)** PS-containing acceptor liposomes with PS-containing (black), PI(3)P-containing (red), PI(4)P-containing (green) or PI(5)P-containing (blue) donor liposomes; **(B)** PS-containing donor liposomes with PS-containing (black), PI(3)P-containing (red), PI(4)P-containing (green) or PI(5)P-containing (blue) acceptor liposomes. Control donor liposome mimics cytoplasmic leaflet of the plasma membrane (23 mol% POPS, 31 mol% POPC, 23 mol% POPE, 23 mol% DHE), and control acceptor liposome mimics ER membrane (5 mol% POPS, 15 mol% liver-PI, 70 mol% POPC, 7 mol% POPE, 3 mol% Dansyl-PE). For PIP-containing liposomes, 2 mol% anionic lipids (POPS and Liver-PI) were replaced by the same amount of PI(3)P, PI(4)P or PI(5)P. Experiments were conducted in HK buffer (50 mM HEPES, 120 mM potassium acetate, pH 7.2) at 37 °C. 100 µM donor and 100 µM acceptor liposomes were incubated with 1 µM StarD4 from time zero. Data are plotted from the average of three independent experiments ± SE. Statistical significance results are shown *, $p < 0.05$; **, $p < 0.01$; ***, $p < 0.001$; ****, $p < 0.0001$; ns, non-significant.

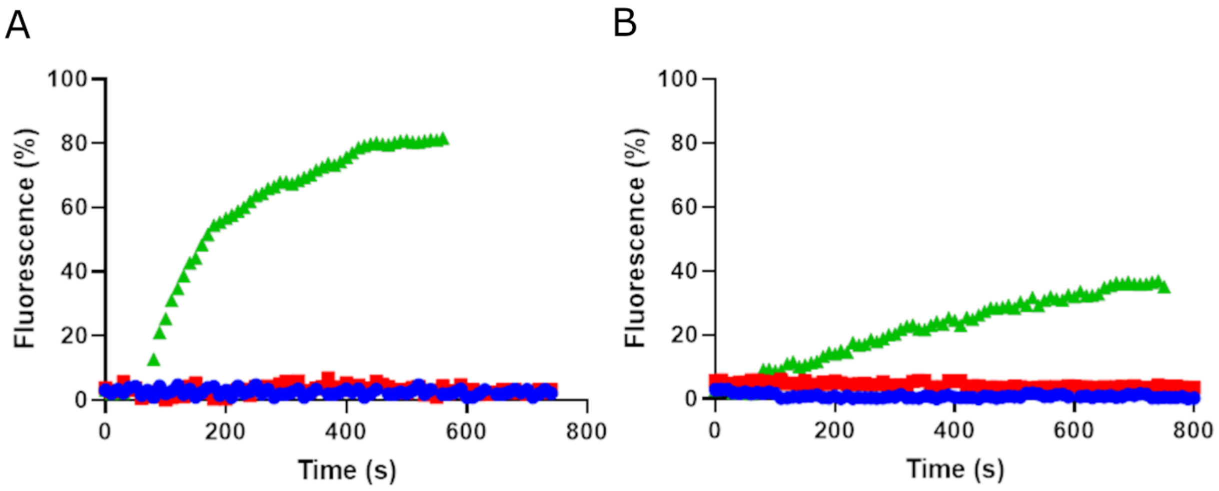

Supporting Figure 4. StarD4 does not induce liposome fusion under experimental conditions. Time course of % change in NBD fluorescence was measured with an excitation/emission wavelength of 465nm/534 nm. Plasma membrane mimic liposomes **(A)** and ER mimic liposomes **(B)** were incubated with 1µM StarD4 (red), 50mM $CaCl_2$ (green), and w/o protein as baseline (blue)**.** Labeled liposomes contained 0.5 mol % NBD-DOPE and 1 mol % Rho-DOPE. The background sample contained only 1 mol % Rho-DOPE. For FRET experiments, 33 µM labeled liposomes were mixed with 167 µM unlabeled liposomes. The NBD fluorescence after adding concentrated $CaCl_2$ to induced fusion is plotted as a positive control.

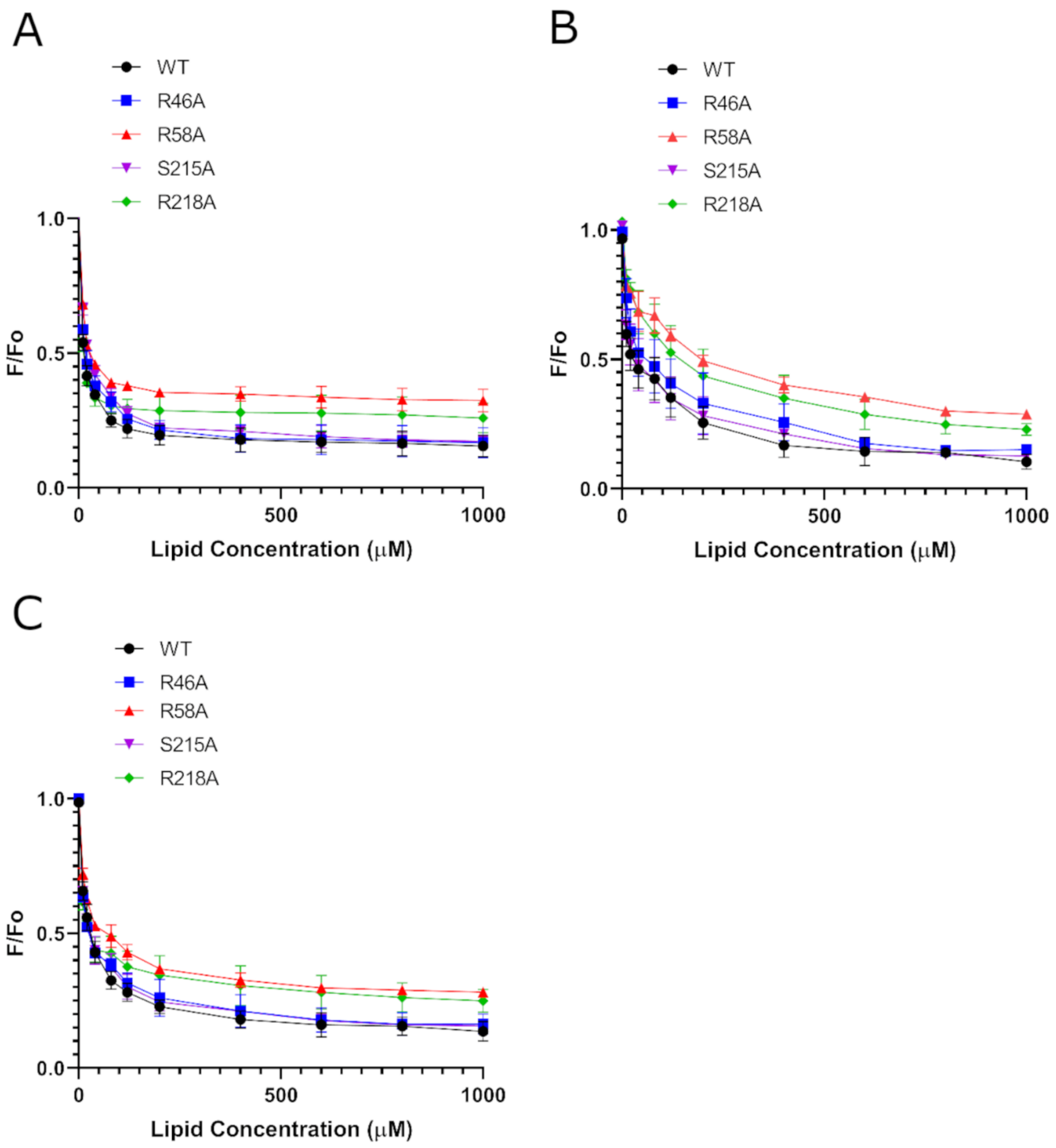

Supporting Figure 5. The initial binding between liposomes and various StarD4 mutants are different. WT and mutants StarD4 were incubated with **(A)** PS-containing, **(B)** PI(4,5)P_2-containing or **(C)** PI(3,4)P_2-containing plasma membrane-mimic liposomes. Plots of tryptophan fluorescence emission at 305 nm vs lipid concentration are displayed. Samples are excited at 270 nm. Increasing amounts of concentrated liposomes were titrated into 2 μM StarD4 solution. Fluorescence was read one minute after titration. F samples contain 23 mol% DHE and Fo samples contain 23 mol% cholesterol. Experiments were conducted in HK buffer (50 mM HEPES, 120 mM potassium acetate, pH 7.2) at 37 °C. Data are plotted from the average of three independent experiments ± SE.

Supporting Table 1. Summary of statistical significance for PIP_2 concentration dependent study. Various mol% of PI(4,5)P_2 or PI(3,5)P_2 were added in donor or acceptor liposomes as previously described.

	**Multiple comparisons test**	**P Value**
**In Donor**	0.5 mol% PI(4,5)P_2 vs 0.5 mol% PI(3,5)P_2	0.99
	1 mol% PI(4,5)P_2 vs 1 mol% PI(3,5)P_2	0.99
	2 mol% PI(4,5)P_2 vs 2 mol% PI(3,5)P_2	0.10
	5 mol% PI(4,5)P_2 vs 5 mol% PI(3,5)P_2	0.0024
	10 mol% PI(4,5)P_2 vs 10 mol% PI(3,5)P_2	<0.0001
**In Acceptor**	0.5 mol% PI(4,5)P_2 vs 0.5 mol% PI(3,5)P_2	0.83
	1 mol% PI(4,5)P_2 vs 1 mol% PI(3,5)P_2	>0.99
	2 mol% PI(4,5)P_2 vs 2 mol% PI(3,5)P_2	0.98
	5 mol% PI(4,5)P_2 vs 5 mol% PI(3,5)P_2	0.89
	10 mol% PI(4,5)P_2 vs 10 mol% PI(3,5)P_2	0.78

Supporting Table 2. Summary of statistical significance for StarD4 mutagenesis study.

(A) comparing effects of StarD4 mutants

	**Multiple comparisons test**	**P Value**
**PS to PS**	WT vs. R46A	<0.0001
	WT vs. R58A	<0.0001
	WT vs. S215A	<0.0001
	WT vs. R218A	<0.0001
**PI(4,5)P_2 to PS**	WT vs. R46A	<0.0001
	WT vs. R58A	<0.0001
	WT vs. S215A	<0.0001
	WT vs. R218A	<0.0001
**PI(3,5)P_2 to PS**	WT vs. R46A	0.046
	WT vs. R58A	<0.0001
	WT vs. S215A	<0.0001
	WT vs. R218A	<0.0001
**PS to PI(4,5)P_2**	WT vs. R46A	<0.0001
	WT vs. R58A	<0.0001
	WT vs. S215A	<0.0001
	WT vs. R218A	<0.0001
**PS to PI(3,5)P_2**	WT vs. R46A	<0.0001
	WT vs. R58A	<0.0001
	WT vs. S215A	<0.0001
	WT vs. R218A	<0.0001
**PI(4,5)P_2 to PI(3,5)P_2**	WT vs. R46A	<0.0001
	WT vs. R58A	<0.0001
	WT vs. S215A	<0.0001
	WT vs. R218A	<0.0001
**PI(3,5)P_2 to PI(4,5)P_2**	WT vs. R46A	<0.0001
	WT vs. R58A	<0.0001
	WT vs. S215A	<0.0001
	WT vs. R218A	<0.0001

(B) comparing effects of PIP in donor or acceptor liposomes

	**Multiple comparisons test**	**P Value**
**WT**	PS to PS vs. PI(4,5)P_2 to PS	<0.0001
	PS to PS vs. PI(3,5)P_2 to PS	0.087
	PS to PS vs. PS to PI(4,5)P_2	0.0003
	PS to PS vs. PS to PI(3,5)P_2	0.013
	PS to PS vs. PI(4,5)P_2 to PI(3,5)P_2	<0.0001
	PS to PS vs. PI(3,5)P_2 to PI(4,5)P_2	<0.0001
**R46A**	PS to PS vs. PI(4,5)P_2 to PS	0.0010
	PS to PS vs. PI(3,5)P_2 to PS	0.33
	PS to PS vs. PS to PI(4,5)P_2	0.91
	PS to PS vs. PS to PI(3,5)P_2	0.59
	PS to PS vs. PI(4,5)P_2 to PI(3,5)P_2	0.96
	PS to PS vs. PI(3,5)P_2 to PI(4,5)P_2	0.18
**R58A**	PS to PS vs. PI(4,5)P_2 to PS	0.79
	PS to PS vs. PI(3,5)P_2 to PS	0.11
	PS to PS vs. PS to PI(4,5)P_2	0.99
	PS to PS vs. PS to PI(3,5)P_2	0.39
	PS to PS vs. PI(4,5)P_2 to PI(3,5)P_2	0.98
	PS to PS vs. PI(3,5)P_2 to PI(4,5)P_2	0.64
**S215A**	PS to PS vs. PI(4,5)P_2 to PS	0.046
	PS to PS vs. PI(3,5)P_2 to PS	0.0065
	PS to PS vs. PS to PI(4,5)P_2	0.44
	PS to PS vs. PS to PI(3,5)P_2	0.0069
	PS to PS vs. PI(4,5)P_2 to PI(3,5)P_2	0.93
	PS to PS vs. PI(3,5)P_2 to PI(4,5)P_2	0.072
**R218A**	PS to PS vs. PI(4,5)P_2 to PS	0.25
	PS to PS vs. PI(3,5)P_2 to PS	0.38
	PS to PS vs. PS to PI(4,5)P_2	0.19
	PS to PS vs. PS to PI(3,5)P_2	0.31
	PS to PS vs. PI(4,5)P_2 to PI(3,5)P_2	0.78
	PS to PS vs. PI(3,5)P_2 to PI(4,5)P_2	0.95

Supporting Table 3. Summary of statistical significance for StarD4 mutagenesis study.

(A) comparing effects of StarD4 mutants

	**multiple comparisons test**	**P Value PI(3)P**	**P Value PI(4)P**	**P Value PI(5)P**
**PS to PS**	WT vs. R46A	<0.0001	0.0001	<0.0001
	WT vs. R58A	<0.0001	<0.0001	<0.0001
	WT vs. S215A	<0.0001	0.0003	<0.0001
	WT vs. R218A	<0.0001	<0.0001	<0.0001
**PIP to PS**	WT vs. R46A	0.0071	<0.0001	<0.0001
	WT vs. R58A	<0.0001	<0.0001	<0.0001
	WT vs. S215A	0.19	0.0009	<0.0001
	WT vs. R218A	0.0013	<0.0001	<0.0001
**PS to PIP**	WT vs. R46A	0.018	<0.0001	<0.0001
	WT vs. R58A	<0.0001	<0.0001	<0.0001
	WT vs. S215A	0.52	0.0027	<0.0001
	WT vs. R218A	0.042	<0.0001	<0.0001

(B) comparing effects of PIP in donor or acceptor liposomes

	**Multiple comparisons test**	**P Value for PI(3)P**	**P Value for PI(4)P**	**P Value for PI(5)P**
**WT**	PS to PS vs. PIP to PS	0.011	0.0021	0.65
	PS to PS vs. PS to PIP	0.021	0.0006	>0.99
	PIP to PS vs. PS to PIP	0.96	0.45	0.65
**R46A**	PS to PS vs. PIP to PS	0.12	0.77	0.33
	PS to PS vs. PS to PIP	0.52	0.28	0.093
	PIP to PS vs. PS to PIP	0.61	0.67	0.37
**R58A**	PS to PS vs. PIP to PS	0.99	0.98	0.92
	PS to PS vs. PS to PIP	>0.99	0.88	0.83
	PIP to PS vs. PS to PIP	0.99	0.96	0.98
**S215A**	PS to PS vs. PIP to PS	0.0041	0.46	0.82
	PS to PS vs. PS to PIP	0.0026	0.78	0.62
	PIP to PS vs. PS to PIP	0.98	0.85	0.94
**R218A**	PS to PS vs. PIP to PS	0.77	0.63	0.94
	PS to PS vs. PS to PIP	0.88	0.54	0.54
	PIP to PS vs. PS to PIP	0.98	0.98	0.74

Supporting Table 4. Summary of statistical significance for membrane environment study.

	**Multiple comparisons test**	**P Value**
WT	PM to ER vs. PM to PM	0.089
	PI(4,5)P2-PM to ER vs. PI(4,5)P2-PM to PM	0.98
	PM to PI(4,5)P2-ER vs. PM to PI(4,5)P2-PM	<0.0001
R46A	PM to ER vs. PM to PM	0.98
	PI(4,5)P2-PM to ER vs. PI(4,5)P2-PM to PM	>0.99
	PM to PI(4,5)P2-ER vs. PM to PI(4,5)P2-PM	0.0045
R58A	PM to ER vs. PM to PM	>0.99
	PI(4,5)P2-PM to ER vs. PI(4,5)P2-PM to PM	>0.99
	PM to PI(4,5)P2-ER vs. PM to PI(4,5)P2-PM	0.97
S215A	PM to ER vs. PM to PM	0.99
	PI(4,5)P2-PM to ER vs. PI(4,5)P2-PM to PM	0.99
	PM to PI(4,5)P2-ER vs. PM to PI(4,5)P2-PM	0.036
R218A	PM to ER vs. PM to PM	>0.99
	PI(4,5)P2-PM to ER vs. PI(4,5)P2-PM to PM	>0.99
	PM to PI(4,5)P2-ER vs. PM to PI(4,5)P2-PM	0.81
